# Fine-Tuned Functionalizable Terpolymer Brush Nanocoating Resists Protein Adsorption and Bacterial Adhesion while Promoting Macrophage Activity and Osteoblast Proliferation

**DOI:** 10.1021/acsami.5c13698

**Published:** 2025-11-20

**Authors:** Alina Pilipenco, Guruprakash Subbiahdoss, Michala Forinová, Oleksandr Romanyuk, Milan Houska, Monika Spasovová, Carmelo Covato, Andrea Scheberl, Erik Reimhult, Hana Vaisocherová-Lísalová

**Affiliations:** † FZU-Institute of Physics of the Czech Academy of Sciences, Na Slovance 1999/2, Prague 182 00, Czech Republic; ‡ Faculty of Mathematics and Physics, Charles University, Ke Karlovu 3, Prague 121 16, Czech Republic; § Institute of Colloid and Biointerface Science, BOKU University, Muthgasse 11, Vienna A-1190, Austria

**Keywords:** biomaterials, polymer brush, antifouling coatings, cell–surface interactions, surface modifications

## Abstract

Developing multifunctional biomaterial surfaces that resist biofouling while supporting cellular activity remains a significant challenge in biomedical engineering. We present a finely tuned functionalizable terpolymer brush nanocoating composed of zwitterionic carboxybetaine methacrylamide (CBMAA), sulfobetaine methacrylamide (SBMAA) and nonionic *N*-(2-hydroxypropyl) methacrylamide (HPMAA) with balanced antifouling cytocompatible characteristics through optimized surface hydration and charge. We analyzed chemical composition, thickness, *ζ*-potential, and wettability using X-ray photoelectron spectroscopy, infrared spectroscopy, spectroscopic ellipsometry, electrokinetic analysis, and water contact angle measurements. Systematic monomer ratio tuning identified poly­(CBMAA 20 mol %-*co*-HPMAA 77 mol %-*co*-SBMAA 3 mol %) as the optimal composition, reducing protein adsorption by 98% in serum-rich media and suppressing *Staphylococcus epidermidis* and *Pseudomonas aeruginosa* adhesion by 99%, effectively preventing biofilm formation under both static and flow conditions. Furthermore, macrophages exhibit enhanced mobility on terpolymer coatings due to their high hydration and low protein adsorption, underpinning reduced adverse immune response. Postfunctionalization with Gly-Arg-Asp (RGD) peptides enabled the adhesion of osteoblast-like SaOS-2 cells while maintaining antifouling efficacy. The tunable multifunctionality of terpolymer brushes in resisting fouling, promoting macrophage phagocytic activity, and supporting SaOS-2 cell adhesion makes them suitable for both antifouling applications and medical implants requiring host tissue integration.

## Graphical abstract

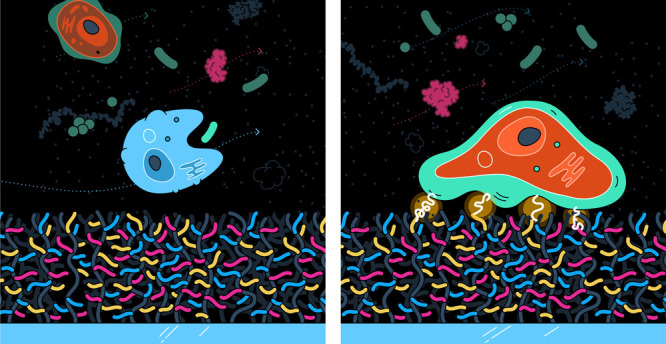

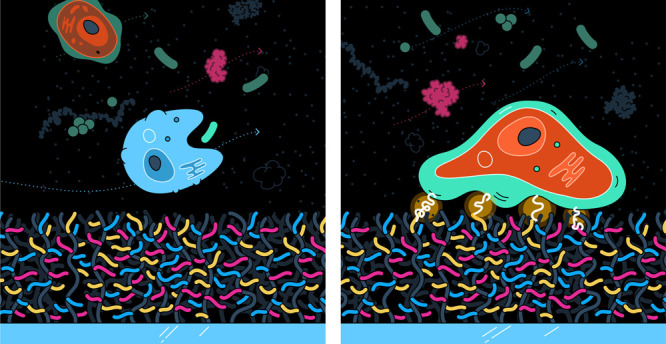

## 1 Introduction

Developing multifunctional biomaterial surfaces remains a major challenge in biomedical applications, where achieving both biocompatibility and resistance to microbial fouling is essential.^1^ When biomaterials come into contact with biological fluids such as plasma or interstitial fluid, their surfaces rapidly adsorb proteins, which alter their physicochemical properties and control interactions with surrounding tissues and microorganisms.^2^ Although protein adsorption can sometimes facilitate integration with the biological environment,^3^ it can also trigger a cascade of adverse effects, including immune activation,^4^ chronic inflammation,^5^ fibrosis,^6^ blood clot formation,^7^ and bacterial adhesion,^8^ ultimately leading to biofilm formation and biomaterial-associated infections (BAIs).^9^ BAIs are frequently caused by opportunistic pathogens such as *Staphylococcus epidermidis* (*S. epidermidis*), *Staphylococcus aureus* (*S. aureus*), *Pseudomonas aeruginosa* (*P. aeruginosa*), *Escherichia coli* (*E. coli*), and *Candida albicans* (*C. albicans*).^10^ Once these bacteria adhere, they establish biofilms by secreting protective extracellular polymeric substances (EPS), forming matrices that shield bacteria from antibiotics and immune responses, making eradication difficult. This problem is further compounded by antimicrobial resistance, which increases both mortality and healthcare costs.^11^

For biomaterial surfaces to be truly effective, they must integrate multiple properties simultaneously: resist protein adsorption and bacterial adhesion, support favorable immune cell responses, and promote biocompatibility to ensure proper tissue integration. Macrophages play a crucial role in the response to implanted biomaterials, managing inflammation, foreign body responses, and microbial defense. Their interactions with surface coatings, especially in a contaminated environment, provide valuable insight into how effectively these coatings support immune clearance in vivo*.* ^12,13^ Beyond pathogen clearance, macrophages contribute to tissue healing and integration by releasing cytokines and growth factors that resolve inflammation and promote regeneration^14–16^ and remodel the extracellular matrix (ECM) – i.e., a process vital for maintaining the stability and functionality of biomedical surfaces.^12,17^ However, some microbes can evade macrophage clearance and form biofilms on biomaterial implants or surrounding tissues.^18^ Additionally, persistent macrophage adhesion to biomaterial surfaces can trigger prolonged inflammation, potentially causing implant rejection or failure.^19^ To optimize biomaterial performance, surfaces should be designed to enhance macrophage mobility and phagocytic activity while preventing excessive adhesion, ensuring efficient immune clearance, and supporting tissue regeneration.

The requirements for biomaterials vary significantly with their intended application. For temporary implant or medical device applications such as urinary catheters, the primary focus is on preventing protein adsorption, which leads to microbial adhesion and biofilm formation, and undesired integration with host cells. In contrast, for the long-lasting or permanent medical devices—such as artificial heart valves, vascular stents, and orthopedic or dental implants—it is imperative not only to prevent microbial adhesion but also to support host cell attachment, proliferation, and differentiation for long-term functionality.^20^ To address these challenges, surface postmodification strategies have been extensively explored, including polymer coatings that mimic the natural extracellular matrix.^21^ Many surface features require optimization, e.g., nanoscale topography, mechanical properties, and surface wettability all regulate cell behavior, including adhesion and proliferation.^22,23^ Additionally, achieving charge neutrality and improving hydration through the balance of cationic and anionic groups is critical in biomaterial design.^24^

Antifouling coatings that prevent protein adsorption and bacterial adhesion remain central to tailoring biointerfaces and are crucial for numerous biomedical applications. Designs of antifouling coatings rely on a variety of building blocks spanning from ethylene glycol derivatives,^25^ zwitterionic,^26,27^ mixed-charged^28^ or nonionic moiety-based self-assembled (nano)­structures as polymer brushes^29–31^ or hydrogels^32,33^ to even complex macromolecular architectures. These also include amphiphilic polymers,^34^ polyethylene glycol (PEG)-like coatings,^35^ poly­(ethylenimine) coatings,^36^ polyelectrolyte multilayer coatings^37^ and zwitterionic metal-phenolic networks,^38^ as well as bottlebrush-like coatings that combine antifouling and antimicrobial properties.^39^ While many of these coatings have shown antifouling efficiency, they are not equally suited for in vivo applications.

To promote cell adhesion while maintaining antifouling properties, biomaterial coatings must possess cell-adhesion motifs, such as Gly-Arg-Asp (RGD) peptides. It is convenient to introduce such sequences through postmodification via functional groups, allowing each coating to be precisely tailored for a specific biomedical application. However, a key challenge lies in achieving sufficient densities of bioactive moieties, such as peptides or growth factors, without compromising the material’s resistance to nonspecific fouling.^40,41^ Studies have demonstrated that clickable polymer brushes and supramolecular elastomers functionalized with RGD peptides can effectively promote cell adhesion while preserving antifouling properties.^42,43^ Furthermore, a recent study suggested that combining antimicrobial agents with RGD peptides in polymer brushes reduces biofilm formation and enhances tissue integration without compromising fouling resistance.^44^ The above studies highlight the potential of tailored surface chemistries to support cell-specific interactions while maintaining antifouling properties against bacteria.

While many coatings have been designed to address one or two of these aspects, a comprehensive approach effectively uniting nonadhesive performance against bacteria, host tissue cell attachment, and immune modulation within a single nanomaterial remains unrealized. The same is valid for our recently reported new terpolymer brush architecture. The terpolymer brush nanocoating is composed of sulfobetaine methacrylamide (SBMAA), carboxybetaine methacrylamide (CBMAA), and *N*-(2-hydroxypropyl) methacrylamide (HPMAA) that maintains long-term stability^45^ and resistance to protein fouling from diverse media, including blood plasma and nasopharyngeal samples,^46^ swab samples from exposed surfaces in the public transport system,^47^ and untreated food samples.^48,49^ This random terpolymer architecture leverages synergistic monomer functionalities: hydrophilic CBMAA introduces zwitterionic carboxylate groups convenient for biofunctionalization, SBMAA provides pH-stable zwitterionic groups for charge balance, and HPMAA establishes a nonionic, hydration-rich backbone. All three building units of the terpolymer effectively contribute to its excellent resistance to fouling. Although this terpolymer brush nanocoating has been demonstrated as an innovative biosensor coating, its biocompatibility, e.g., its compatibility with human cells and its interactions with host immune cells, remains largely unexplored. Nevertheless, very promising results on cytocompatibility for polymer brushes having similar structures were reported. Beyond protein fouling resistance, initial studies on homo- and binary copolymer systems (e.g., poly­(CBMAA*-co-*HPMAA)) have revealed that cellular interactions are highly sensitive to brush composition—specifically, higher content of CBMAA promoted cell spreading and actin fiber formation, while increased HPMAA led to reduced spreading and more rounded morphologies, highlighting the important role of zwitterionic moieties in modulating cell behavior.^50^ These findings suggest that even subtle structural variations in polymer coatings may significantly impact biological performance, yet a comprehensive understanding of these effects is not fully explained.

In this study, we present a finely tuned, functionalizable terpolymer brush nanocoating designed for versatile biomedical applications. This poly­(SBMAA-*co*-CBMAA-*co*-HPMAA) terpolymer brush enables precise control over surface hydration, charge repulsion, and protein adsorption through adjustments in monomer ratios that are practically realizable and can be tailored for different biomaterial applications. In addition to optimizing antifouling properties against both protein and bacterial adhesion, we introduce a comprehensive evaluation framework in which the brushes were assessed for bacterial clearance by macrophage activity, and for osteoblast proliferation. Thereby representing both host defense and tissue integration. We demonstrate that the fouling resistance of the coating can be modulated by altering the CBMAA content, which directly affects surface hydration and charge density; further, it provides functional groups for subsequent biofunctionalization, which we exploit for RGD peptide coupling. The key biological metrics, including protein adsorption, bacterial adhesion and biofilm growth, osteoblast-like cell attachment and spreading, macrophage responses, and bacterial phagocytosis, were investigated to elucidate how subtle structural modifications impact the biological performance of these polymer brush coatings. Our findings will substantially impact the design of next-generation biomaterials that effectively balance antifouling efficiency with biological compatibility across diverse biomedical settings.

## 2 Results and Discussion

### 2.1 Tailoring Zwitterionic Brushes for Tunable Charge and Biofunctionality

Surface-initiated atom transfer radical polymerization (SI-ATRP) was employed to fabricate robust antifouling binary copolymer and terpolymer brushes composed of CBMAA (CB), HPMAA (HP), and SBMAA (SB) (Figure 1). SI-ATRP was chosen because it provides precise control over brush composition and thickness, which is essential for systematically investigating structure-function relationships in these coatings. Although the method requires rigorous deoxygenation, it enables reproducible initiator chemistry and remains the benchmark for mechanistic studies in fundamental research. For future translation to scaled synthesis and industrial applications, air-tolerant ATRP variants such as ARGET ATRP,^51^ ICAR ATRP,^52^ and SI-PET-RAFT^53,54^ could be advantageous, as they reduce oxygen sensitivity or eliminate the use of heavy metal catalysts. Three distinct compositions-pHP_(97)_SB_(3)_ (binary copolymer), pCB_(20)_HP_(77)_SB_(3),_ and pCB_(30)_HP_(67)_SB_(3)_ (terpolymers)-were synthesized by fixing the SBMAA content at 3 mol % and systematically adjusting the CBMAA content from 0 mol % to 30 mol % to investigate the impact of the density of carboxybetaine monomers, providing functionalizable anionic groups. Limiting CBMAA to a maximum of 30 mol % avoids an overabundance of the carboxyl reactive groups that could compromise antifouling performance while providing a sufficient number of groups for subsequent biofunctionalization.^55^ This strategy offers an effective means to assess how carboxyl surface density influences the balance between antifouling capability and reactivity.

**Figure 1:**
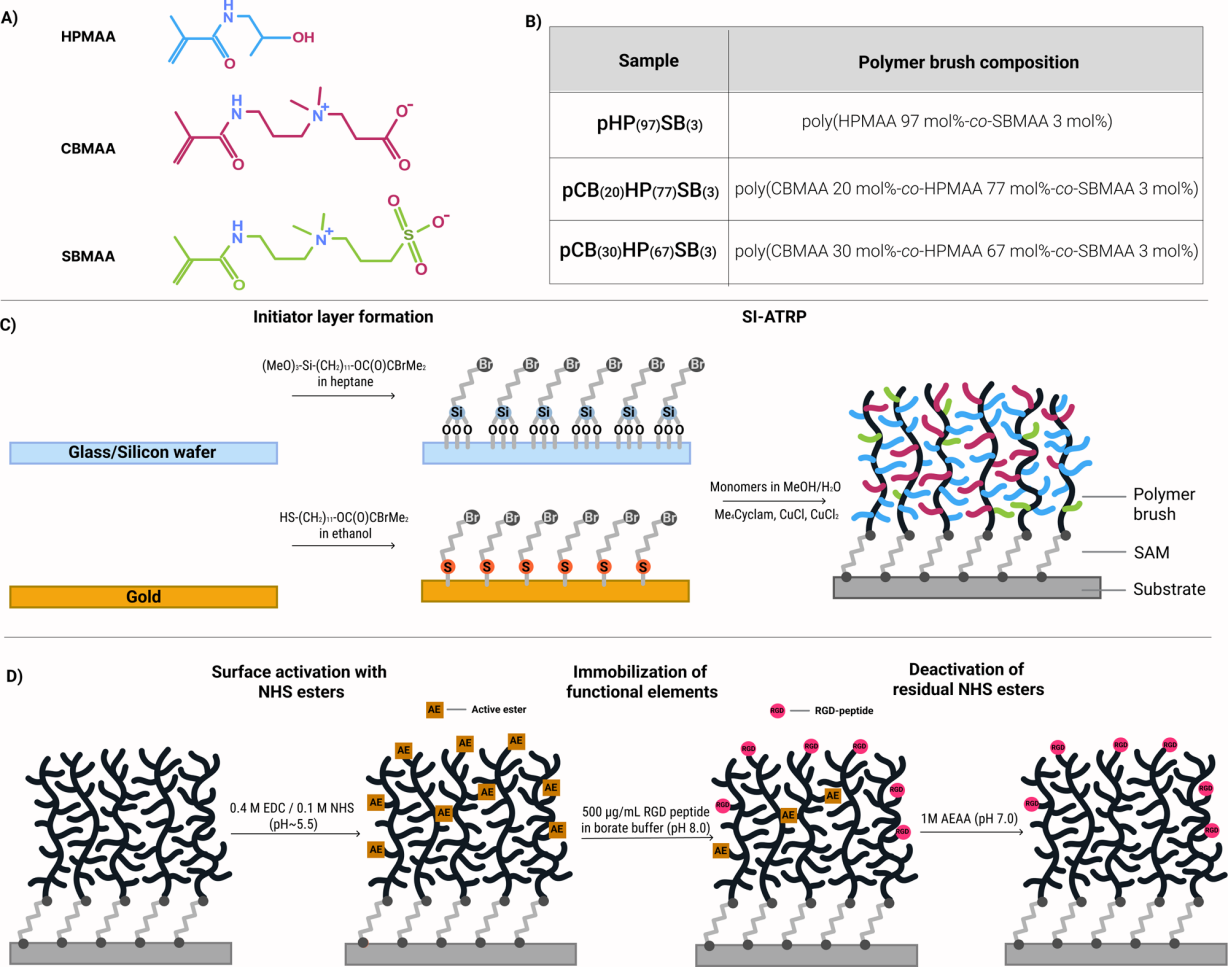
Schematic representation of (A) the chemical structures of monomers, (B) the investigated polymer brush compositions, (C) illustration of the polymer brush synthesis via the “grafting-from” SI-ATRP technique on different substrates, and (D) bioconjugation of polymer brush coatings with RGD peptide using 1-ethyl-3-(3-(dimethylamino)­propyl) carbodiimide (EDC)/*N*-hydroxysuccinimide (NHS) chemistry.

Random copolymerization of the CBMAA, HPMAA, and SBMAA monomers resulted in polymer brushes with a thickness of 27 ± 3 nm in the dry state and 66 ± 12 nm in water (swelling ratio *α* ∼2.4), as measured by ellipsometry on gold-coated substrates (Table S1). The swelling ratio is related to the surface density of hydrophilic polymer chains; a higher swelling ratio usually indicates a lower surface density.^56^ Although the composition of the coatings varies, their swelling ratios are quite similar (Table S1), suggesting that their surface densities are also comparable.

X-ray photoelectron spectroscopy (XPS) and infrared grazing angle attenuated total reflection spectroscopy (IR-GAATR) confirmed the successful polymerization of all studied polymer brush compositions on the substrates (Figure 2). XPS measurements showed increased C, N, and decreased Si concentrations compared to bare glass (Table S2), and the presence of expected functional groups on the surface. The fit of quaternary ammonium (C–N^+^) peak bonds increased with higher CBMAA content (Figure 2A,B), confirming controlled incorporation of CBMAA in the polymer brush. The N 1s peak analysis revealed that CBMAA and SBMAA concentrations agreed with the corresponding concentrations in the polymerization feed within an error bar of 5% (Figure 2C). The homogeneity of the polymer brush composition was further confirmed by angle-resolved (AR) XPS measurements and simulations, which confirmed no dependence of core level spectral line shapes and composition on emission angle (i.e., information depth) for the pCB_(30)_HP_(67)_SB_(3)_ sample (Figure S1). The IR-GAATR spectra of polymer brushes on silicon wafers reveal prominent amide I (1640 cm^–1^) and II (1540 cm^–1^) bands. The band of ionized carboxyl groups (COO^–^) is seen at 1610 cm^–1^ overlapped with the amide I band and a very weak broad band at 1733 cm^–1^ indicates nonionized carboxyl groups (−COOH) (Figure 2D). pSBMAA generally exhibits two sulfo (-SO^3–^) bands at 1215 cm^–1^ and 1040 cm^–1^. However, the first band overlaps with the intense SiO_2_ band of the substrate and the second band is weak due to the low concentration of SBMAA (3 mol %) in the terpolymer. The presence of SBMAA in the polymer brushes is supported by XPS (Table S2), where sulfur was detected at concentrations below 1 at. % - significantly lower than in the SBMAA monomer (5.1 at. %), which is consistent with the low SBMAA content in the terpolymer (3 mol %); the corresponding S 2p signal (Figure S1), though weak, confirms successful incorporation and complements the IR data, where band overlap limited unambiguous identification.

**Figure 2:**
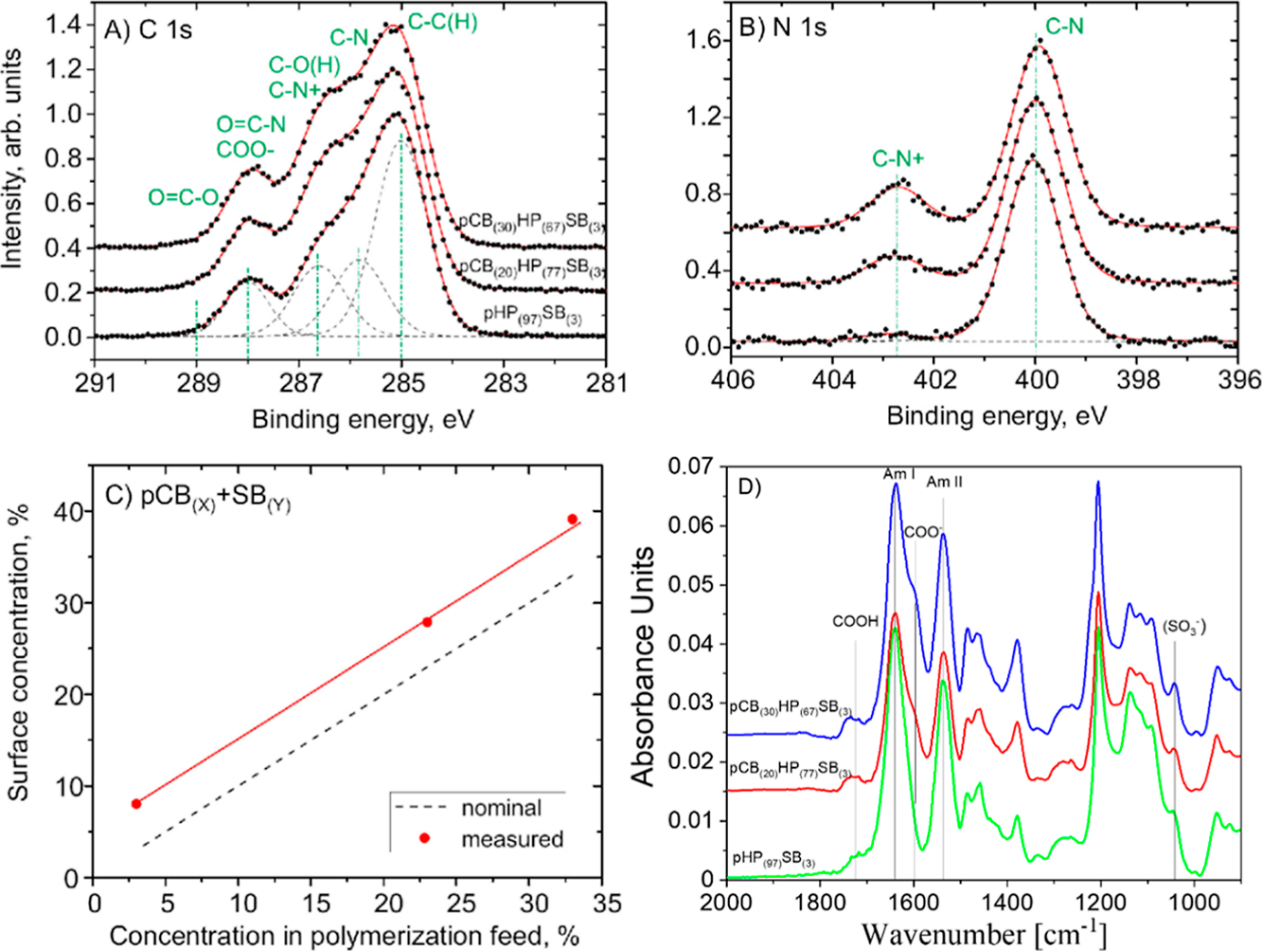
High-resolution (A) C 1s and (B) N 1s XPS spectra of samples. Symbols and lines indicate data points and fits, respectively. C 1s contains contributions from C–C­(H), C–N, C–O­(H), protonated C–N^+^, O=C–N, and COO^–^ bonds. N 1s peaks showed contributions from C–N and C–N^+^ bonds. (C) Concentrations of CBMAA and SBMAA monomers in samples derived from N 1s peak intensities. The dashed line represents the monomer concentration in the polymerization feed, while the symbols indicate the derived CBMAA + SBMAA surface atomic concentration. (D) IR-GAATR spectra of polymer brushes on a silicon wafer.

Dynamic water contact angle measurements showed reduced advancing (*θ*_a_) and receding (*θ*_r_) angles on the coated glass compared to bare glass (Table S3). Receding angles decreased systematically with higher CBMAA content, resulting in increased contact angle hysteresis (Δ*θ* = *θ*_a_ – *θ*_r_). The elevated hysteresis for brushes correlates with enhanced surface hydration via zwitterionic carboxylate–water interactions. CBMAA’s –COO^–^ groups retain interfacial water through electrostatic forces (lowering *θ*_a_), while creating energy barriers to dewetting during receding measurements (limiting *θ*_r_). This aligns with our previous study of carboxylate-rich zwitterionic systems^57^ where structured interfacial water reduces dewetting efficiency.

Noncoated glass substrates are characterized by highly negative surface *ζ*-potentials of around −40 mV.^58^ When coated with the brushes investigated in this paper, the magnitude of the negative *ζ*-potential was reduced to −13 mV for pCB_(20)_HP_(77)_SB_(3)_ and −23 mV for pCB_(30)_HP_(67)_SB_(3)_ at physiological pH (7.4) (Table S4). A similar trend was observed for pCB and pSB homopolymer brushes grown from gold-coated substrates.^59^ However, it was observed that the interaction of pCB and pSB brushes, which exhibit negative *ζ*-potential values, with polycations at physiological pH was relatively weak. Therefore, caution is warranted when interpreting absolute *ζ*-potential values of zwitterionic polymer brush nanocoatings, and greater emphasis should be placed on the observed trends.^59^

To verify the RGD immobilization conditions, we tested brushes grafted onto gold chips, using surface plasmon resonance (SPR), and we detected an RGD density of 51 ng/cm^2^ on pCB_(20)_HP_(77)_SB_(3)_ (Figure S2). To further corroborate successful NHS-EDC-derived conjugation, we observed a significant shift in *ζ*-potential and characteristic changes in the IR spectra. Specifically, IRRAS measurements revealed a decrease in the intensity of bands attributed to ionized carboxyl groups (1610 and 1365 cm^–1^), indicating their consumption during the coupling reaction. Additionally, subtle broadening in the amide I region (∼1690 cm^–1^) and increased absorption in the fingerprint region (1230–1080 cm^–1^) were observed, consistent with the presence of peptide-specific vibrational modes (Figure S3). The conjugation of the RGD peptide to the terpolymer brushes was performed at pH 8.0, which is above their pIs, so the positively charged RGD peptide should be attracted to the surface, or at least not be repelled. The immobilization of the positively charged RGD peptide resulted in a *ζ*-potential shift for both terpolymer brushes to less negative values, i.e. from −13.4 mV to −2.7 mV for pCB_(20)_HP_(77)_SB_(3)_ and from −23.5 mV to −7.1 mV for pCB_(30)_HP_(67)_SB_(3)_. The “blank-pCB_(20)_HP_(77)_SB_(3)_” surface, which was subjected to the same immobilization conditions but in the absence of RGD peptide, showed only negligible changes in *ζ*-potential, confirming that the observed changes can be properly attributed to the RGD immobilization.

### 2.2 Impact of Brush Composition on Protein Adsorption

Protein adsorption on biomaterial surfaces is a major factor determining their performance in vivo. The adsorption of various proteins can either inhibit further physiological responses or produce detrimental effects, such as enhanced platelet adhesion and thrombus formation.^60^ We evaluated the antifouling properties of the polymer brushes with respect to protein adsorption using quartz crystal microbalance with dissipation monitoring (QCM-D). The measurements were conducted in Dulbecco’s Modified Eagle Medium with low glucose (DMEM-LG) supplemented with 10% fetal bovine serum (FBS), a commonly used cell culture medium that contains proteins, such as albumin, fibronectin, and growth factors. These proteins are known to strongly foul material surfaces, making this medium a suitable choice for testing resistance to protein adsorption.

As shown in Figure 3, the brush coatings exhibited remarkable resistance to protein adsorption before and after RGD peptide immobilization, significantly (*p* < 0.01) reducing adsorption compared to the bare gold surfaces (Figure 3). Protein adsorption on the pCB_(30)_HP_(67)_SB_(3)_ was reduced by an average of 83.2% compared to bare gold crystal. The pHP_(97)_SB_(3)_ exhibited an average of 96.8% reduction in protein adsorption. Among the tested compositions, the terpolymer containing pCB_(20)_HP_(77)_SB_(3)_ demonstrated the best performance, reducing the protein adsorption to 12.6 ± 1.6 ng/cm^2^, giving a ∼98% reduction. The coupling of RGD peptide further improved the fouling resistance. Although in our recent paper,^59^ we did not find a correlation between negative *ζ*-potential values and polycation adsorption, it is noteworthy that the immobilized positively charged RGD peptide shifted the *ζ*-potential closer to zero (Table S4) and thus could reduce double-layer-driven adsorption. Representative changes in the sensor response of the gold-coated sensor crystal surface with or without the polymer brush coatings exposed to the protein-rich medium are presented in Figure S4.

**Figure 3:**
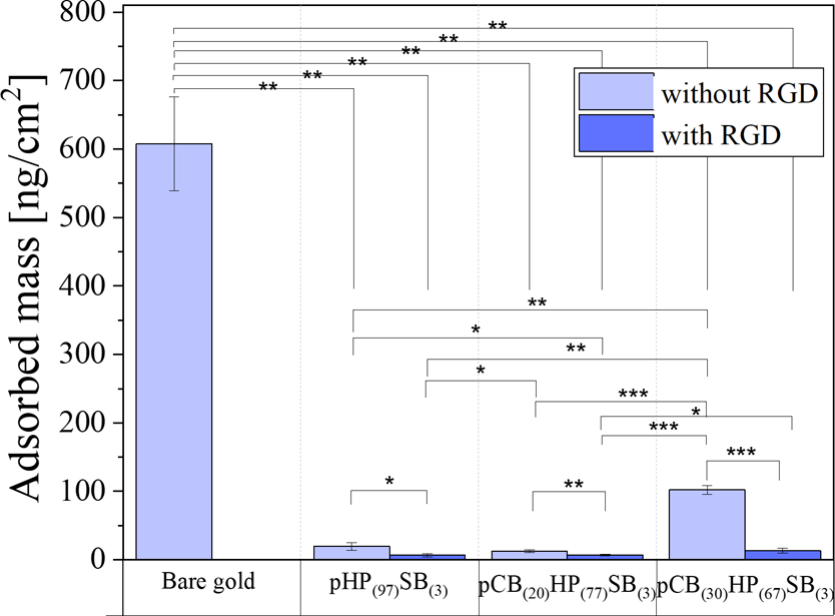
Protein adsorption on the three polymer brushes from DMEM-LG supplemented with 10% FBS was measured using QCM-D. Adsorbed mass was calculated from Δ*f*/n using the Sauerbrey equation for the 3rd overtone for polymer brush-coated and bare gold QCM crystals. Error bars represent the standard deviation from at least three measurements. Significant differences are indicated by * = *p* ≤ 0.05; ** = *p* ≤ 0.01, *** = *p* ≤ 0.001.

A direct quantitative comparison of the fouling resistance of zwitterionic brushes from literature data is difficult due to many factors like different polymerization techniques, substrates, test media compositions and methods for fouling measurement and evaluation. It is recognized that factors such as the degree of polymerization (chain length) and the grafting density of the brush play essential roles.^61^ Unlike QCM-D, which measures the “wet” adsorbed mass, including water added to the adsorbed layer, optical methods such as SPR predominantly capture the “dry” mass. The adsorbed mass recorded by QCM-D can, therefore, be expected to be several times higher for proteins at low surface coverage.^62^ For example, a previous study on carboxy-based terpolymer brushes reported 5.3 ng/cm^2^ for undiluted plasma using SPR.^46^ Pérez et al.,^31^ observed 17 ng/cm^2^ of protein adsorption in diluted human serum on polyphosphoester polymer brushes using QCM-D. Studies on zwitterionic polymers and coatings consistently report low protein adsorption values, and insights from these studies provide a broader context for evaluating the pCB_(20)_HP_(77)_SB_(3)_ brush. (Table S5). In this context, the adsorbed hydrated mass of 13 ng/cm^2^ determined from DMEM-LG supplemented with FBS using QCM-D in very good agreement.

### 2.3 Tuning Zwitterionic Brush Coatings to Suppress Bacterial Adhesion and Biofilm Formation

The resistance of brush coatings against bacterial adhesion was examined through a set of complementary bacterial adhesion experiments. The strongly biofilm-forming opportunistic pathogens *S. epidermidis* ATCC 35984 (Gram-positive) and *P. aeruginosa* PAO1 (Gram-negative) were flowed through a microfluidic chamber, with coated or noncoated glass coverslips as the bottom plate, for 2 h. Testing both types of bacteria provided more comprehensive insight into bacterial interactions with the coatings due to the distinct surface structural and charge characteristics of Gram-positive and Gram-negative bacteria.

The polymer brush coatings significantly inhibited *P. aeruginosa* and *S. epidermidis* adhesion after 2 h compared to the noncoated glass coverslips (Figures 4 and S5). The pHP_(97)_SB_(3)_ reduced *P. aeruginosa* adhesion by 98.3% (log reduction of 1.8) and for *S. epidermidis* by 98.9% (log reduction of 1.9). The pCB_(30)_HP_(67)_SB_(3)_ polymer brush surface demonstrated a 95.1% reduction for *P. aeruginosa* (log reduction of 1.3) and a 98.7% reduction for *S. epidermidis* (log reduction of 1.9). The most effective reduction was observed with pCB_(20)_HP_(77)_SB_(3)_, achieving a 99.0% reduction for *P. aeruginosa* (log reduction of 2.0) and a 99.3% reduction for *S. epidermidis* (log reduction of 2.2). Bacterial adhesion correlated with the surfaces’ resistance to protein adsorption.

**Figure 4:**
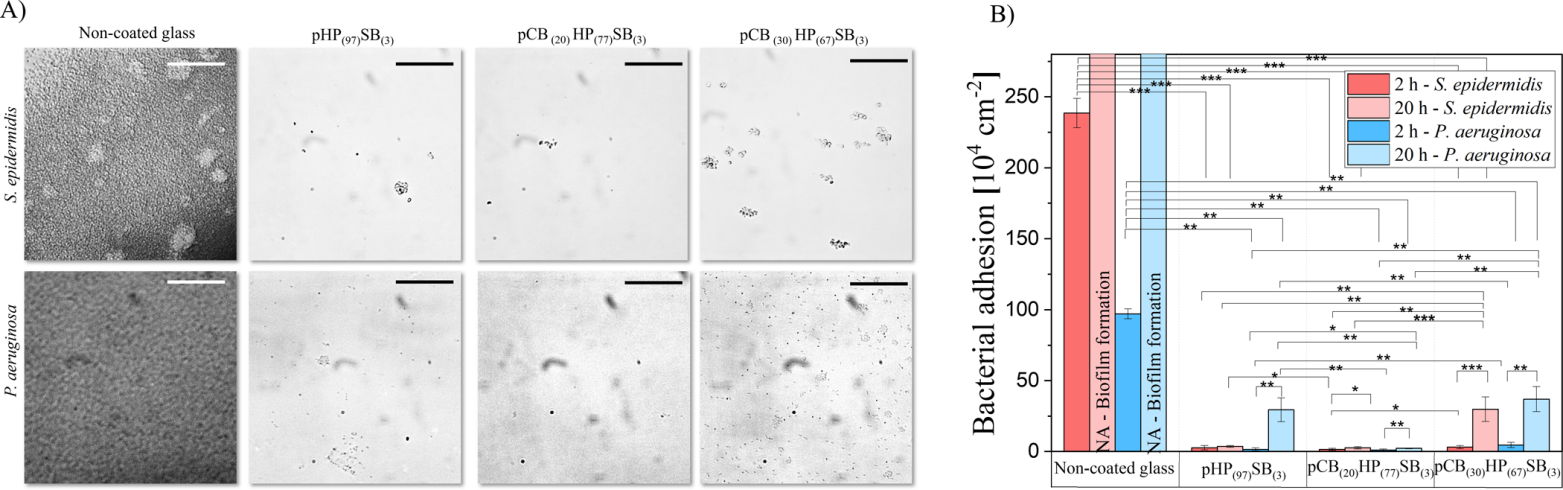
(A) Representative bright field microscopic images of bacterial biofilm formation on polymer-coated and noncoated glass after 20 h of growth. (B) Number of adherent *S. epidermidis* (red) and *P. aeruginosa* (blue) after an initial adhesion of 2 h and after 20 h of growth in a microfluidic chamber on coated and noncoated glass. Error bars represent the standard deviation from at least three technical replicates. Significant differences are indicated by * = *p* ≤ 0.05; ** = *p* ≤ 0.01; *** = *p* ≤ 0.001.

To assess biofilm formation, the study was extended to 20 h in a continuous flow of sterile tryptic soy broth (TSB) medium, maintained at 37 °C. Complete biofilm formation was evident on noncoated glass after 20 h (Figure 4A). In contrast, pCB_(20)_HP_(77)_SB_(3)_ suppressed the biofilm formation of *P. aeruginosa* and *S. epidermidis*, maintaining adherent cell densities of (2.3 ± 0.3) × 10^4^ and (2.7 ± 0.8) × 10^4^ bacteria/cm^2^, respectively. Similar long-term antifouling performance of pCB_(20)_HP_(77)_SB_(3)_ was confirmed in static conditions after 24 h of growth in TSB at 37 °C (Figures S6 and S7). In comparison, pCB_(30)_HP_(67)_SB_(3)_ showed the highest bacterial counts among the polymer-coated surfaces (Figure 4B). Consequently, pCB_(30)_HP_(67)_SB_(3)_ was not selected for long-term experiments, as its antifouling performance under physiological flow conditions was lower than for the other compositions.

Following the RGD coupling, bacterial adhesion on pHP_(97)_SB_(3)_ and pCB_(20)_HP_(77)_SB_(3)_ remained significantly lower than on the noncoated glass (Figure S8). For *S. epidermidis*, RGD-functionalized polymer brushes showed slightly higher adhesion - (3.9 ± 0.9) × 10^4^ and (3.1 ± 0.6) × 10^4^ bacteria/cm^2^ for pHP_(97)_SB_(3)_ and pCB_(20)_HP_(77)_SB_(3)_, respectively - compared to nonfunctionalized ones - (2.7 ± 1.5) × 10^4^ and (1.6 ± 0.7) × 10^4^ bacteria/cm^2^. Despite these small increases, the adhesion was reduced by approximately 98% on both coatings compared to noncoated glass (2.4 × 10^6^ bacteria/cm^2^). This limited response to RGD peptide is likely due to the overall near-neutral polymer brush ζ-potential and the bacteria’s thick peptidoglycan layer, which restricts direct interactions between surface-bound RGD peptides and bacterial teichoic acids. Additionally, the hydration-mediated repulsion within the zwitterionic brush layer may help counteract cationic RGD-driven bacterial interactions. In contrast, *P. aeruginosa* showed a more substantial increase in adhesion following RGD functionalization, rising 12.4-fold on pHP_(97)_SB_(3)_ ((1.6 ± 1.1) × 10^4^to (20.3 ± 1.7) × 10^4^ bacteria/cm^2^) and 7.9-fold on pCB_(20)_HP_(77)_SB_(3)_ ((1.0 ± 0.9) × 10^4^ to (7.7 ± 2.4) × 10^4^ bacteria/cm^2^). Nevertheless, adhesion was still reduced compared to noncoated glass (9.7 × 10^5^ bacteria/cm^2^). We attribute the higher adsorption of *P. aeruginosa* compared to *S. epidermidis* mainly to its more negative surface potential due to its outer membrane containing negatively charged lipopolysaccharides, which interact more strongly with the positively charged RGD peptides. Furthermore, *P. aeruginosa*’s motility and ability to bind at a distance from the surface, aided by pili and flagella, may allow it to exploit minor binding sites on the modified surfaces, overcoming some of the antifouling effects. Additionally, physical adsorption of RGD peptides directly to the underlying substrate surface could also contribute to minor bacterial adhesion, as peptides may penetrate the polymer brush layers and form new positively charged interaction sites at defects of the polymer coating.^63^

The stable brush structure of pCB_(20)_HP_(77)_SB_(3)_ provided consistent antifouling properties, outperforming other tested compositions. This performance highlights the critical role of surface characteristics, particularly zwitterionic balance, wettability, steric hindrance, and coating stability, in minimizing bacterial adhesion via minimizing protein fouling. Following RGD peptide immobilization, pCB_(20)_HP_(77)_SB_(3)_ showed only a slight decrease in antifouling performance compared to the nonfunctionalized surface, with a 7% reduction in efficacy against *P. aeruginosa* (from 99.0% to 92.0%) and a 0.6% reduction for *S. epidermidis* (from 99.3% to 98.7%), demonstrating adaptability for functionalization without compromising performance. The hydrophilic zwitterionic nature of pCB_(20)_HP_(77)_SB_(3)_ ensured minimal bacterial growth during the incubation period, contributing to long-term antifouling performance across various conditions, including static environments and under physiologically relevant shear stress, which are critical for maintaining surface stability over extended periods. By maintaining stable brush architecture and minimizing attractive interactions with proteins and bacteria, the terpolymer coating effectively prevents the cascade of events leading to biofilm formation.

While comparing results across studies is inherently challenging due to differences in bacterial strains, incubation times, and experimental methods, the performance of pCB_(20)_HP_(77)_SB_(3)_ (3 × 10^4^ bacteria/cm^2^) aligns with and outperforms the efficacy demonstrated for similar zwitterionic coatings (Table S6). For instance, Cheng et al.,^64^ observed no biofilm formation for pSBMAA surfaces exposed to *S. epidermidis* and *P. aeruginosa* after 24 h. But compared to carboxybetaine functionalized polysiloxanes reported by Cheng et al.,^65^ yielded *E. coli* adhesion of 10^5^ bacteria/cm^2^ after 24 h, pCB_(20)_HP_(77)_SB_(3)_ demonstrates a major improvement. Similarly, Liu et al.,^66^ observed adhesion levels around 10^4^ bacteria/cm^2^ after 24 h of cultivation of *S. epidermidis* and *P. aeruginosa* on amino acid-based zwitterionic polymers. While previous studies report low bacterial adhesion (see Table S6) on zwitterionic polymers, our findings emphasize that compositional tuning enables surfaces to simultaneously resist protein/bacterial fouling and retain programmable biofunctional capacity, balancing hydration-mediated antifouling with tailored bioactivity for diverse biomedical applications.

### 2.4 Effects of Polymer Brush Coatings on J774A.1 Murine Macrophage Activity

We investigated J774A.1 murine macrophage behavior in the presence of *S. epidermidis* on different surface coatings using bright-field microscopy, exposing the surfaces to bacterial adhesion for 15 min prior to macrophage introduction to mimic peri-operative contamination conditions where implants become contaminated during implantation (i.e., during surgery). The number of adherent *S. epidermidis* on polymer brush-coated surfaces was approximately **∼**2 × 10^4^ bacteria/cm^2^, compared to about 1 × 10^6^ bacteria/cm^2^ on noncoated glass. This mimics clinically relevant bacterial contamination levels (ranging from 10^2^ to 10^5^ cells/cm^2^) seen in sterile surgical settings.^13^

Our results showed that macrophages exhibited distinctly different behavior on polymer-brush-coated glass compared to noncoated glass. On noncoated glass, macrophages adopted elongated morphologies (Supporting Information Video si_002), indicative of stable adhesion via integrin-mediated binding to adsorbed proteins (e.g., fibronectin). After phosphate-buffered saline (PBS) wash, noncoated glass coverslips retained a higher density of adhered macrophages (Figure 5A), which could interfere with other essential cell types by inducing inflammatory responses or competing for surface binding sites. This contrasts with polymer brushes, where reduced protein adsorption (Section 2.2) limits ligand availability for adhesion receptors, such as CD11b/CD18. While strong adhesion stabilizes cell attachment, it may reduce macrophage mobility, which could potentially decrease the effectiveness of bacterial clearance.^67^ The macrophages maintained a spherical shape, exhibited enhanced mobility (Supporting Information Videos si_003 and si_004), and were easily washed away with PBS on polymer-brush-coated surfaces (Figures 5A and S9). Real-time microscopy demonstrated that macrophages on polymer-coated surfaces actively engaged with bacteria, efficiently migrating across the surface using pseudopodia to engulf bacteria. In contrast, although the number of macrophages was comparable across surfaces, macrophages on noncoated glass appeared more static and less functionally engaged. It is plausible that the high swelling ratio and water content of polymer brushes enable macrophages to adopt a migratory phenotype. Over 120 min, bacterial surface numbers decreased significantly through macrophage action on all investigated surfaces (Figure 5a), with an average reduction of 56.6% on the noncoated glass coverslips, 50.8% on the pHP_(97)_SB_(3)_, and 56.9% on the pCB_(20)_HP_(77)_SB_(3)_, calculated as (*N*_–M_ – *N*_+M_)/*N*_–M_×100, where *N*_–M_ and *N*_+M_ correspond to bacterial counts in the absence and presence of macrophages. These clearance rates were calculated by comparing bacterial adhesion on surfaces with macrophages to the same surfaces without macrophages under identical incubation conditions in cell culture medium at 37 °C.

**Figure 5:**
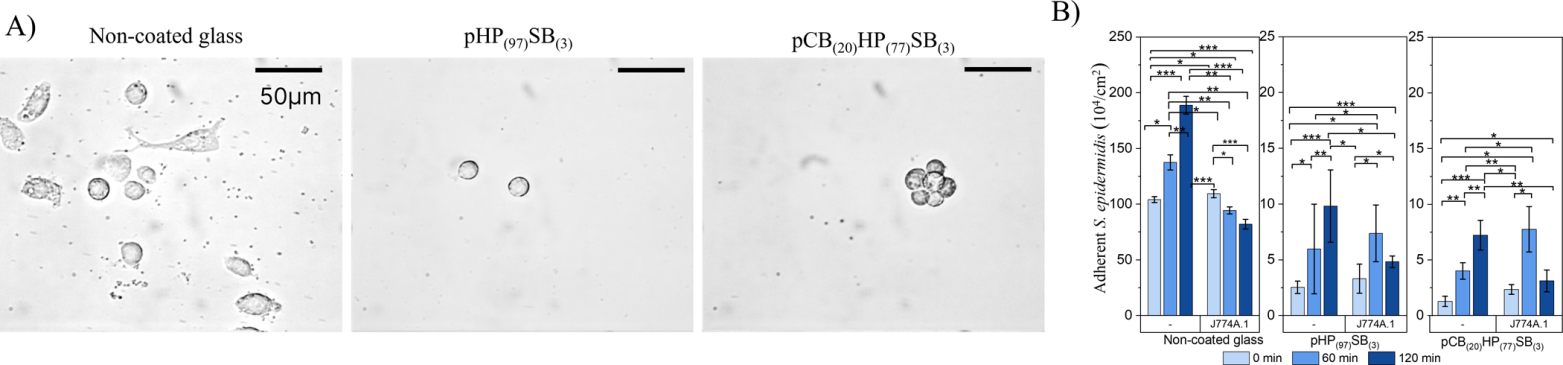
(A) Representative phase-contrast images illustrate the morphologies of J774A.1 murine macrophages and *S. epidermidis* adhering to different coatings after 120 min of coincubation and post-PBS washing to remove loosely bound macrophages and *S. epidermidis*. The scale bar represents 50 μm. (B) Number of adherent *S. epidermidis* on noncoated and coated glass in the absence or presence of J774A.1 murine macrophage over 120 min in serum-containing cell culture media. The phagocytic activity of macrophages is shown by the bacterial adhesion density of *S. epidermidis* on various coatings at 0, 60, and 120 min. Significant differences are indicated by * = *p* ≤ 0.05; ** = *p* ≤ 0.01; *** = *p* ≤ 0.001.

These results demonstrate that macrophages effectively phagocytosed bacteria on noncoated and pCB_(20)_HP_(77)_SB_(3)_ coated glass, while pHP_(97)_SB_(3)_ showed slightly reduced but comparable performance. Although fewer macrophages adhered to the terpolymer surfaces, they were more mobile and exhibited higher phagocytic efficiency per cell. Consequently, these clearance ratios were achieved with several times more active macrophages on noncoated glass surfaces than on the terpolymer brush grafted surfaces, demonstrating the higher phagocytotic efficiency due to mobility on the latter. However, noncoated glass exhibited high bacterial adhesion after 120 min, with macrophage activity only partially reducing the bacterial load. The higher initial bacterial adhesion on noncoated glass likely provided macrophages with more targets, amplifying the observable clearance effect. In contrast, the reduced bacterial adhesion on polymer brushes limits bacterial proliferation and ensures prolonged antifouling performance.

Previous studies have demonstrated that surface chemistry, charge, and topography strongly influence macrophage behavior and macrophage–bacterial interactions. Saldarriaga Fernández et al.,^13^ showed that PEG-based coatings enhanced macrophage motility and bacterial clearance (∼70%) but still permitted biofilm formation over time. Similarly, da Silva Domingues et al.,^68^ found that patterned PEG-hydrogels improved macrophage phagocytosis, while cationic polyethyleneimine (PEI) coatings restricted macrophage mobility, limiting their ability to clear bacteria. Park and Bryers^12^ demonstrated that IFN-γ and LPS-functionalized PEG coatings activated macrophages to kill bacteria more efficiently but at the cost of reduced phagocytosis efficiency, increasing the risk of excessive immune activation. Additionally, da Silva Domingues et al.,^69^ reported that hydrophobic glass surfaces increased macrophage phagocytosis but also facilitated bacterial adhesion and biofilm formation.

Unlike these previously studied surfaces, pCB_(20)_HP_(77)_SB_(3)_ successfully balances antifouling properties with macrophage mobility, preventing bacterial colonization while allowing macrophages to remain active without signs of impaired viability or excessive clustering. By tuning the polymer composition, such as incorporating CBMAA and adjusting brush hydration, the coatings effectively reduce macrophage adhesion while maintaining their mobility. These findings highlight the potential of polymer brush coatings on biomaterials that strike the right balance by reducing bacterial adhesion while simultaneously supporting macrophage mobility and phagocytosis to reduce BAIs.

### 2.5 Balancing Cellular Adhesion with Antifouling Efficiency for Permanent and Temporary Biomaterials

In follow-up studies with SaOS-2 osteosarcoma cells, all polymer brush coatings used in this work exhibited strong resistance to SaOS-2 cell attachment in their initial unmodified state (Figure 6), with pCB_(20)_HP_(77)_SB_(3)_ showing minimal attachment (0.6 × 10^3^ cells/cm^2^). After 24 h of incubation under flow (5 μL/min) in a microfluidic chamber, only a few SaOS-2 cells adhered to polymer brush-coated surfaces, displaying a rounded morphology with membrane blebbing, a sign of apoptotic cell death (Figure S10). This underscores the utility of these materials for temporary implants, e.g., urinary catheters, where resistance to host cell attachment, bacterial adhesion, and biofilm formation is desirable for minimizing inflammation or infection during application and facilitating removal after use.

**Figure 6:**
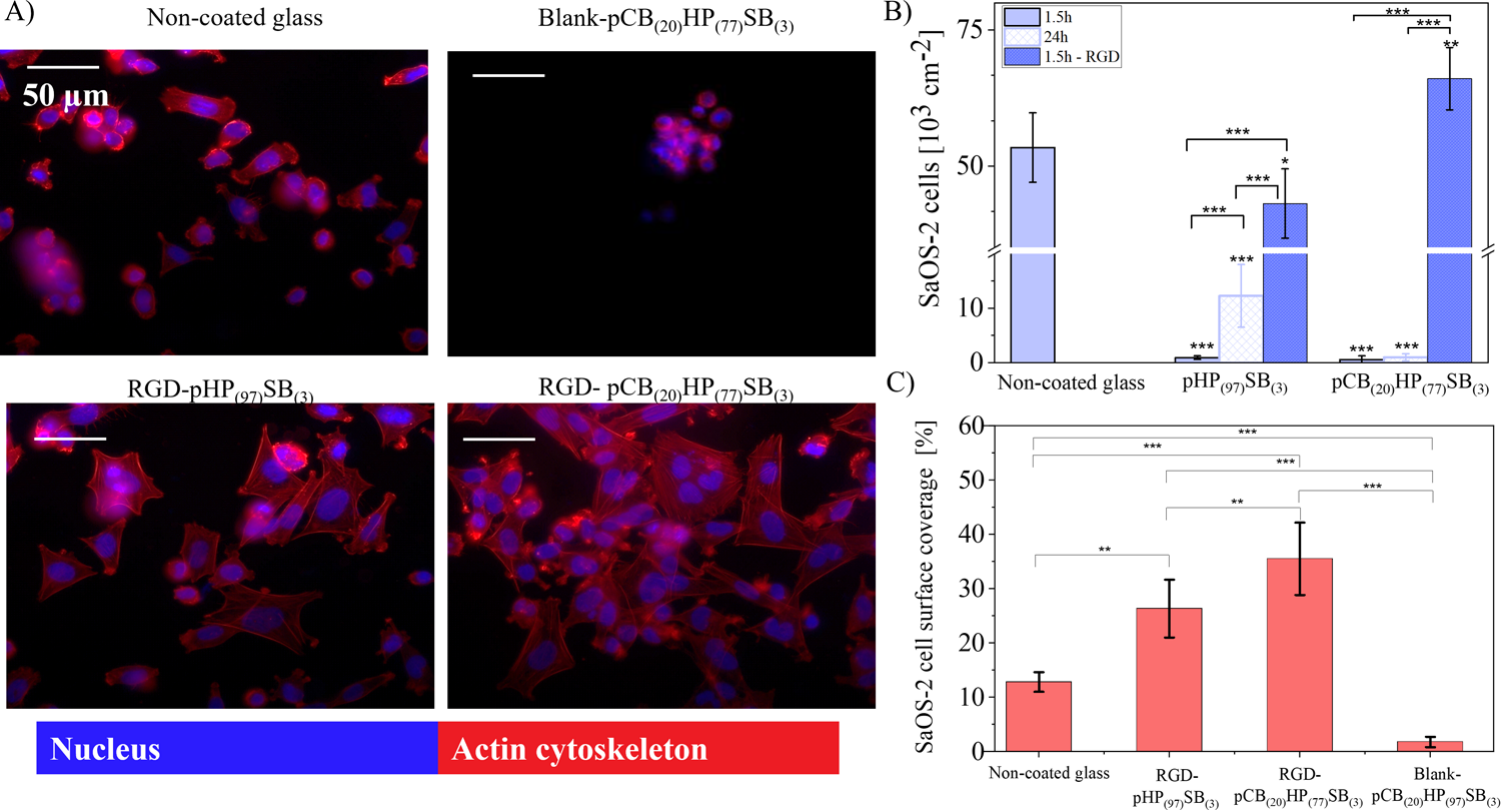
SaOS-2 cell adhesion and proliferation with or without RGD peptide. (A) Representative fluorescence microscopy images of SaOS-2 cells stained after 1.5 h of cultivation on the coated and noncoated glass surfaces. (B) SaOS-2 cell adhesion on noncoated glass coverslips, binary copolymer pHP_(97)_SB_(3)_ and terpolymer pCB_(20)_HP_(77)_SB_(3)_ brush surfaces at 1.5 h and 24 h, with/without RGD peptide. (C) SaOS-2 cell surface coverage on noncoated glass coverslip, RGD-pHP_(97)_SB_(3)_, RGD-pCB_(20)_HP_(77)_SB_(3)_, and blank-pCB_(20)_HP_(77)_SB_(3)_ (subjected to RGD peptide binding procedure in RGD peptide absence) at 1.5 h. Significant differences are indicated by * = *p* ≤ 0.05; ** = *p* ≤ 0.01; *** = *p* ≤ 0.001.

However, effective cellular adhesion and tissue formation are crucial for permanent or long-term biomaterials, such as orthopedic or dental implants, which are intended to integrate with bone or soft tissues. Functionalization with RGD peptides, which are known to promote integrin binding, makes antifouling brushes cell-adhesive. At 1.5 h of incubation, RGD-functionalized polymer brush surfaces dramatically increased SaOS-2 cell adhesion (Figures 6 and S11), from 0.6 × 10^3^ cell/cm^2^ to 66.1 × 10^3^ cell/cm^2^, showing an approximate 117-fold improvement. This effect was observed even on polymer brush surfaces without CBMAA (pHP_(97)_SB_(3)_)_,_ suggesting physisorption of RGD inside the brush despite thorough rinsing, as discussed in Section 2.3.

While the total number of SaOS-2 cells on noncoated glass after 1.5 h appeared comparable to the polymer-coated surfaces, the surface coverage, calculated based on the area with visible actin cytoskeleton from fluorescence images, revealed that cells on the polymer brush surfaces exhibited greater spreading (Figure 6C). For pHP_(97)_SB_(3)_ exposed to the RGD, cell surface coverage increased to 26.4%, more than doubling the control (12.9%). With the RGD-functionalized pCB_(20)_HP_(77)_SB_(3),_ the surface coverage reached 35.5%, nearly tripling that of the noncoated glass (12.9%). Figure 6C further demonstrates that SaOS-2 cells on RGD-functionalized surfaces had well-spread morphology with a pronounced actin cytoskeleton and nuclei, indicating robust cell attachment and spreading. A previous study demonstrated that increasing the concentration of RGD peptides in poly­(carboxy-betaine acrylamide) (pCBAA) promoted enhanced cell growth and spreading.^70^ Moreover, increasing the CBMAA content in binary copolymer brushes enhanced nuclear localization of Yes-associated protein (YAP), which correlated with increased cell spreading.^70^

In contrast, SaOS-2 cells on nonfunctionalized polymer brushes appeared less spread and more clustered, indicating an unfavorable environment for cell adhesion and proliferation. Moreover, on the blank-pCB_(20)_HP_(77)_SB_(3)_ (no RGD added during the bioconjugation procedure), cell spreading was minimal with only 1.8% surface coverage (Figure 6), with cells behaving similarly to those on nonfunctionalized polymer brushes. These observations demonstrate that covalent RGD coupling via CBMAA carboxyl groups was critical for achieving selective cell adhesion without compromising antifouling performance.

## 3 Conclusions

Our study demonstrates the rational and systematic integration of antifouling, suppression of bacterial adhesion, inducible osteoblast adhesion, and immune cell motility and bacterial clearance activity within a single tunable polymer brush system, which is still awaiting a practical and adjustable realization. By tuning the composition of CBMAA, HPMAA, and SBMAA monomer units, we establish a versatile interface balancing antifouling properties with bioactivity for various applications. The optimized poly­(CBMAA 20 mol %-*co*-HPMAA 77 mol %-*co*-SBMAA 3 mol %) composition achieved a 98% reduction in protein adsorption and over 99% reduction in bacterial adhesion compared to noncoated surfaces. The terpolymer brush enhanced macrophage motility and phagocytic activity, facilitating efficient bacterial clearance. RGD peptide functionalization significantly improved osteoblast-like cell adhesion and spreading, achieving a 117-fold increase in cellular attachment while maintaining resistance to proteins and bacterial fouling, and further improving phagocyte surface motility and activity.

Our comprehensive evaluation highlights the importance of compositional adjustments in achieving a multifunctional biomaterial surface. Unlike previous studies that examine these factors in isolation, our integrated approach provides a holistic understanding of how polymer brush coatings interact with multiple biological systems, leading to a more complete assessment of their multifunctionality. The integration of zwitterionic, hydrophilic, and reactive monomers enables formulating coatings that resist nonspecific fouling while supporting selective cell interactions when required. The stoichiometrically tunable nature of this system provides a flexible and adaptable platform for diverse biomedical applications, from anti-infective coatings for temporary implants and biomedical devices to permanent implants requiring host tissue integration. Our study provides crucial insights into the structure–function relationships of polymer brush coatings, establishing a foundation for the rational design of next-generation biomaterials.

## 4 Experimental Section

### 4.1 Preparation of Polymer Brushes on Substrates

Gold-coated quartz crystals (5 MHz, Q-Sense AB, Sweden), glass coverslips (Ibidi GmbH, Germany), silicon wafers (Siegert Wafer GmbH, Germany), and SPR chips were used. Gold-coated substrates were sonicated in ethanol, rinsed with Milli-Q water, dried under nitrogen, and activated via UV–ozone for 20 min. Glass and silicon substrates were sonicated in acetone, treated with 50% isopropanol, and activated similarly. Functionalization of glass and silicon involved immersion in a 1 mM solution of (MeO)_3_–Si-(CH_2_)_11_-OC­(O)­CBrMe_2_ in heptane, while gold substrates were treated with 1 mM HS-(CH_2_)_11_-OC­(O)–CBrMe_2_ in ethanol to form self-assembled monolayers (SAMs). Polymer brushes were synthesized via SI-ATRP as previously described,^48^ in three compositions: poly­(HPMAA 97 mol %*-co-*SBMAA 3 mol %); poly­(CBMAA 20 mol %*-co-*HPMAA 77 mol %*-co-*SBMAA 3 mol %); poly­(CBMAA 30 mol %*-co-*HPMAA 67 mol %*-co-*SBMAA 3 mol %) (Figure 1). After polymerization, substrates were rinsed with ultrapure water and stored in PBS (pH 7.4) at 4 °C. Polymer brush thickness was measured using spectroscopic ellipsometry,^50^ surface wettability via dynamic water contact angle measurements,^57^ and *ζ*-potential via an electrokinetic analyzer.^58^

### 4.2 RGD Functionalization of Polymer Brushes

Substrates were sterilized in 70% ethanol (30 min), rinsed, and activated with 0.1 M NHS and 0.4 M EDC for 20 min. RGD peptide (500 μg/mL, Biosynth, USA) in 10 mM borate buffer (pH 8) was immobilized for 20 min, followed by deactivation with 1 M aminoethoxy acetic acid (30 min). Coatings were rinsed, dried under nitrogen, and stored for further use. RGD immobilization was monitored in real time by SPR using a BioNavis multiparametric SPR instrument (4-channel microfluidics). After baseline stabilization in water, chips were sequentially exposed to NHS/EDC, RGD solution, and rinsing steps, and the angular response was converted to surface mass density.

### 4.3 Infrared GAATR and IRRAS Analysis of Polymer Brushes

Infrared grazing angle attenuated total reflectance (GAATR) and infrared reflection–absorption spectroscopy (IRRAS) spectra were acquired on a Nicolet iS50 FTIR spectrometer using VariGATR (63°) and Smart SAGA (80°) accessories, from 200 scans at 4 cm^–1^ resolution.

### 4.4 X-ray Photoelectron Spectroscopy (XPS)

XPS was performed using a monochromated Al Kα source (1486.6 eV, 150 W), with an electron flood gun for charge neutralization. Core-level spectra were recorded at 0.1 eV steps, and atomic concentrations were calculated via Shirley background subtraction and sensitivity factors (ESCApe software, Kratos).

### 4.5 Protein Adsorption Assay Using QCM-D

QCM-D (Q-Sense AB, Sweden) was used to assess protein adsorption on polymer brushes at 180 μL/min flow rate. Measurements were performed in triplicate, with PBS stabilization (20 min), protein injection (20 min), and PBS washing (20 min) at 25 °C. Mass changes were calculated using the Sauerbrey equation (*C* = 17.7 ng·cm^–2^·Hz^–1^).

### 4.6 Bacterial Cultivation

*S. epidermidis* ATCC 35984 and *P. aeruginosa* PAO1 ATCC 15692 were cultivated in tryptone soy broth (TSB) and lysogeny broth (LB), respectively, at 37 °C, 100 rpm. Bacterial cells were harvested (5000 rpm, 5 min), resuspended in PBS, and adjusted to 0.4 OD at 600 nm for flow and biofilm experiments.

### 4.7 Bacterial Adhesion and Biofilm Growth

Bacterial adhesion was studied using a Nikon Eclipse TE2000 microscope (Nikon Europe B.V, Vienna, Austria). A bottomless, self-adhesive microfluidic structure (sticky-slide I 0.2 mm, Ibidi GmbH, Germany) was glued to coated or noncoated glass coverslips. The microfluidic chamber was filled with sterile PBS and the bacterial suspension was perfused for 2 h at 0.5 mL/min. Loosely bound bacteria were washed out prior to biofilm growth (20 h, 0.35 mL/min, TSB or LB). Images were captured every 5 min for adhesion and every 20 min for biofilm growth. *Static biofilms* were grown by inoculating surfaces with bacterial suspensions (OD = 0.4) in sterile Petri dishes (37 °C, 24 h). Biofilms were imaged using phase-contrast microscopy (40× objective) and stained with LIVE/DEAD (SYTO 9 and propidium iodide, Invitrogen). Image analysis was performed using ImageJ software.

### 4.8 Cell Culture and Adhesion Studies

SaOS-2 osteosarcoma cells (ACC 243, DSMZ) and murine macrophages J774A.1 (DSMZ) were cultured in DMEM/McCoy’s 5A with 15% fetal bovine serum (FBS) and 1% Penicillin–Streptomycin at 37 °C in 5% CO_2_. Cells were detached using TrypLE, resuspended, and counted using a Countess automated cell counter.

### 4.9 Monocyte Migration and Phagocytic Activity

J774A.1 monocyte interactions with *S. epidermidis* were studied microscopically in a microfluidic chamber (Ibidi) at 37 °C. After bacterial priming, 2.5 × 10^6^ monocytes/mL were introduced at 0.5 mL/min. Bright-field images (40× objective) were captured every 1 min for 120 min. Controls included bacterial cultures without monocytes and sterile conditions.

### 4.10 SaOS-2 Cell Attachment and Spreading

SaOS-2 cells (2.5 × 10^6^ cells/mL) were injected into a microfluidic chamber, incubated (37 °C, 90 min), and imaged (20× objective) every 5 min. The medium was refreshed (5 μL/min, 22 h), followed by fixation (Roti Histofix, 10 min), permeabilization (0.5% Triton X-100, 3 min), and staining (DAPI and TRITC-phalloidin, 30 min). Fluorescence microscopy was used for surface coverage analysis.

### 4.11 Statistical Analysis

Data were expressed as means ±standard deviation (*n* ≥ 3). Statistical significance was determined using one-way ANOVA (*p < 0.05, **p < 0.01, ***p < 0.001). For more details, see Supporting Information.

## Supplementary Material









## Data Availability

Data for this article will be available at Zenodo repository (10.5281/zenodo.17415044).

## Notes

Experimental procedures: Preparation and SI-ATRP grafting of polymer brushes; RGD peptide immobilization; Physicochemical surface characterization (IR-GAATR, ellipsometry, contact angle, XPS); QCM-D-based protein adsorption assay; Bacterial culture and biofilm formation protocols; Macrophage and osteoblast (SaOS-2) culturing and interaction assays; Quantitative image-based analysis of cell adhesion and bacterial colonization; Figures S1 to S11: Characterization of brush morphology, composition (XPS, ellipsometry, IR-GAATR), biofilm formation, cell morphology, and fluorescence imaging; Tables S1 to S6: Polymer thickness/swelling, surface composition (XPS), contact angles, *ζ*-potential values, protein adsorption, and bacterial adhesion comparison (PDF)
Legend for Video si_002 (in separate file): Simultaneous incubation of *S. epidermidis* and J774A.1 murine macrophage over 120 minutes on uncoated glass (MP4)
Legend for Video si_003 (in separate file): Simultaneous incubation of *S. epidermidis* and J774A.1 murine macrophage over 120 minutes on pHP_(97)_SB^_(3)_^ (MP4)
Legend for Video si_004 (in separate file): Simultaneous incubation of *S. epidermidis* and J774A.1 murine macrophage over 120 minutes on pCB_(20)_HP_(77)_SB_(3)_ (MP4)
