# Supplementary material for: Fine-Tuned Functionalizable Terpolymer Brush Nanocoating Resists Protein Adsorption and Bacterial Adhesion while Promoting Macrophage Activity and Osteoblast Proliferation
Source: ACS Appl Mater Interfaces. 2025 Nov 20;17(48):65399–410. doi: 10.1021/acsami.5c13698 (PMC12679544; doi:10.1021/acsami.5c13698)
Supplement: Supplementary file 1 [file am5c13698_si_001.pdf]

# Supporting Information

**Fine-Tuned Functionalizable Terpolymer Brush Nanocoating Resists Protein Adsorption and Bacterial Adhesion while Promoting Macrophage Activity and Osteoblast Proliferation**

*Alina Pilipenco[1,2], Guruprakash Subbiahdoss[3], Michala Forinová[1,2], Oleksandr Romanyuk[1], Milan Houska[1], Monika Spasovová[1], Carmelo Covato[3], Andrea Scheberl[3], Erik Reimhult[3,*], Hana Vaisocherová-Lísalová[1,*]*

1 - FZU - Institute of Physics of the Czech Academy of Sciences, Na Slovance 1999/2, 182 00 Prague, Czech Republic
2 - Faculty of Mathematics and Physics, Charles University, Ke Karlovu 3, 121 16 Prague, Czech Republic
3 - Institute of Colloid and Biointerface Science, BOKU University, Muthgasse 11, A-1190 Vienna, Austria

*Corresponding Authors:

Hana Vaisocherová-Lísalová, Ph.D., FZU - Institute of Physics, Academy of Sciences of the Czech Republic, v.v.i., Na Slovance 2, 182 00 Prague, Czech Republic; Tel.: +420 266 05 29 93, E-mail: lisalova@fzu.cz

Univ. Prof. Dr. Erik Reimhult, Institute of Colloid and Biointerface Science, BOKU University, Muthgasse 11, A-1190 Vienna, Austria, Tel.: +43 1 47654 80211, E-mail: erik.reimhult@boku.ac.at

**Contents:**

- Figures S1 to S11
- Tables S1 to S6

# Supplementary Materials and Methods with additional details

## 1. Preparation of Polymer Brushes on Substrates

We utilized 5 MHz gold-coated quartz crystals (Q-Sense AB, Sweden), glass coverslips (25 mm × 75 mm, Ibidi GmbH, Germany), silicon wafers (Siegert Wafer GmbH, Germany), and 12 mm × 20 mm SPR chips for our experiments. The gold-coated quartz crystals were initially cleaned by sonicating in ethanol for 10 min, followed by thorough rinsing with Milli-Q water (18.0 MΩ·cm, Milli-Q system, Merck, Germany), and drying with nitrogen gas. Subsequently, the crystals were activated in a UV-ozone cleaner (Jelight Company, USA) for 20 min. Glass slides and silicon wafers were first rinsed with acetone, then sonicated for 20 min in 50% isopropanol (Sigma-Aldrich, Germany). Subsequently, the substrates were activated in a UV-ozone cleaner for 20 min. Then, glass coverslips and silicon wafers were immediately immersed in a freshly prepared 1 mM solution of $(MeO)_3$-Si-$(CH_2)_{11}$-OC(O)CBr$Me_2$ (ProChimia Surfaces, Poland) in dry heptane (Merck, Germany). Gold-coated substrates were treated in a solution containing 1 mM HS-$(CH_2)_{11}$-OC(O)-CBr$Me_2$ (ProChimia Surfaces, Poland) in ethanol (99.8%, for UV spectroscopy) from Lachner, Czech Republic to create a self-assembled monolayer (SAM) that served as an initiator. The polymer brush coatings were prepared using a modified method based on surface-initiated atom transfer radical polymerization (SI-ATRP), as previously described.[1] These polymer chains were synthesized as a polymer with a random distribution of carboxybetaine methacrylamide (CBMAA), *N*-(2-hydroxypropyl) methacrylamide (HPMAA), and sulfobetaine methacrylamide (SBMAA) (Specific Polymers, France) in three different compositions. Subsequently, the substrates were rinsed with ultra-pure water and stored in phosphate-buffered saline (PBS) solution (0.01 M sodium phosphate, 0.138 M sodium chloride, 0.0027 M potassium chloride, pH 7.4) (Sigma-Aldrich, Germany) in the refrigerator until they were used. The zeta potential of the planar surfaces was determined using an electrokinetic analyzer (SurPass, Anton Paar, Austria) with an adjustable-gap cell, according to the protocol detailed previously.[2]

## *2. RGD-functionalisation of polymer brushes*

Before functionalization, coatings were rinsed with ultrapure water and sterilized in 70% ethanol for 30 min, as previously described.[3] Subsequently, coatings were immersed in ultrapure water for 5 min, activated with a fresh mixture of 0.1 M *N*-hydroxysuccinimide (NHS) (Cytiva, Sweden) and 0.39 M *N*-ethyl-*N*'-(3-dimethylaminopropyl) carbodiimide hydrochloride (EDC) (Cytiva, Sweden) in water for 20 min. Then, the coatings were shortly

washed with ultrapure water, and a solution of 500 μg/mL RGD peptide (H-RRRGGGGRGDSP-OH, Biosynth, USA) in borate buffer (10 mM, pH 8) (Merck, Czech Republic) was added and reacted for 20 min. After immobilization, the coatings were rinsed with MilliQ water and immersed in the deactivation solution of 1 M aminoethoxy acetic acid (AEAA) (AlfaAesar, Germany) for 30 min. Finally, all the coatings were rinsed with PBS, dried with a nitrogen stream, and prepared for the subsequent steps.

RGD immobilization was also monitored in real time by surface plasmon resonance (SPR). SPR experiments were performed using a multiparametric SPR instrument with a 4-channel microfluidic system (BioNavis, Finland) and a 670 nm LED source. The angular response was converted to surface mass density (0.001° ≙ 0.85 ng $cm^{-2}$).[4] Chips were washed, dried, and mounted, baseline was established in water, and then sequential injections of NHS/EDC (20 min, 5 μL/min), water (4 min, 60 μL/min), 50 μg/mL of RGD in borate buffer (20 min, 15 μL/min), and water (10 min, 15 μL/ min), were performed.

## 3. *Infrared Spectroscopic Characterization of Polymer Brushes: Infrared* **Grazing Angle Attenuated Total Reflectance Spectroscopy** *(GAATR) and Infrared Reflection Absorption Spectroscopy (IRRAS)*

The IR-GAATR spectra of polymer brushes on silicon wafers were measured using a Thermo Scientific™ Nicolet™ iS50 FTIR spectrometer (Thermo Fisher Scientific, USA) equipped with a Harrick Scientific VariGATR™ grazing angle accessory. The IRRAS spectra confirming the presence of the RGD-peptide were acquired with the same spectrometer equipped with a Smart SAGA™ accessory (Thermo Scientific™). All spectra were collected from 200 scans at a resolution of 4 $cm^{-1}$. For IRRAS measurements, the beam incident angle was set to 80°, while GAATR measurements were performed at an incident angle of 63°.

## *4. Ellipsometry*

The thickness of polymer coatings was determined from ellipsometric measurements using J.A. Woollam Spectroscopic ellipsometer (Lincoln, USA). Spectra of samples in both dry and wet states were taken in a glass cuvette under $70^0$ incident angle from 300 to 1100 nm with a step of 10 nm. The depolarization did not exceed 2%. The coatings were modelled in WVASE32 software using one oscillator based on a Gaussian distribution.

## *5. Contact angle*

Surface wettability was assessed at room temperature using a DSA100 drop shape analyzer (Krüss GmbH, Germany) via the sessile drop method. A 2 μL water droplet was placed

on the surface, then expanded and reduced by 8 µL at 0.5 µL/s. Advancing and receding contact angles were determined from droplet shape changes using a tangent fitting algorithm.

## *6. X-ray photoelectron spectroscopy (XPS)*

XPS measurements were performed by AXIS Supra photoelectron spectrometer (Kratos) using a monochromated Al Kα source (1486.6 eV, 150 W) focused on a 0.7 × 0.3 $mm^2$ area. Photoelectron intensities were measured normal to the surface at 0°. A pass energy of 10 eV provided an overall energy resolution of 0.53 eV (verified on Ag $3d_{5/2}$). Despite grounding samples by a metallic plate, significant charging was observed, necessitating an electron flood gun for charge neutralization and consequently calibration of the spectra to the position of C 1s peak at 285 eV. Core level spectra were acquired with 0.1 eV steps. Atomic concentrations were quantified by subtracting Shirley backgrounds and applying sensitivity factors (implemented in the ESCApe software, Kratos). Spectral fitting utilized Gauss-Lorentz functions with 0.3 ratio. The C 1s spectra contain C-C/C-H, C-N, overlapping C-O/C-OH/C-$N^+$, O=C-N/COO-, and O=C-O components of the corresponding functional groups. The N1s spectra consists of contributions from C-N and C-$N^+$ bonds. The measured C-$N^+$ / C-N intensity ratio was 0.85 ± 0.10 for the reference CBMAA monomer sample. Utilizing this ratio, we can derive the surface concentration of CBMAA and SBMAA polymers in the samples (note, both CBMAA and SBMAA contain C-$N^+$ bonds, whereas only C-N bonds are present in HPMAA. Measured surface concentrations are present in Fig. 2C in the manuscript.

Angle-resolved XPS spectra are present in Figure S1 A. By variation of emission angle from 0° (normal emission) to an emission angle of 70°, the information depth of XPS was changed from about 10 nm to 2 nm, correspondingly. The line shapes of all core level peaks were preserved for different depths, indicating a highly homogeneous surface. In Figure S1 B, simulated angle-dependent atomic surface concentrations are present. Simulations confirmed the expected atomic composition of $pCB_{(30)}HP_{(67)}SB_{(3)}$ NP with an error bar of a few at.%.

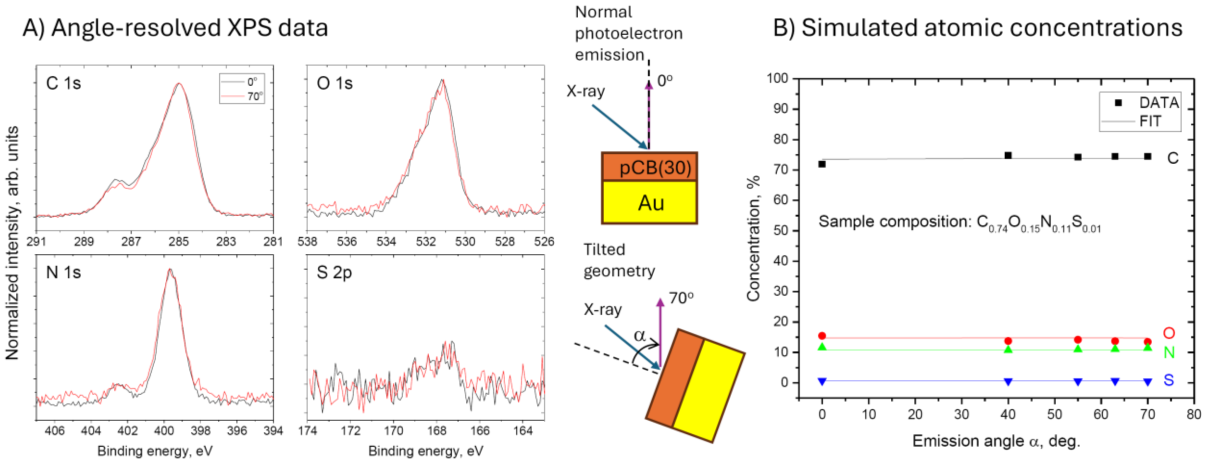


**Figure S1.** A) Angle-resolved XPS core level spectra of C 1s, O 1s, N 1s, and S 2p measured from the pCB$_{(30)}$HP$_{(67)}$SB$_{(3)}$/Au sample with photoelectron emission angles of 0° (normal emission) and 70° (tilted sample, providing more surface-sensitive data). B) Simulated dependence of the polymer brush atomic composition on the emission angle, i.e., at different depths.

## 7. *Protein adsorption assay using QCM-D*

The study utilized Quartz Crystal Microbalance with Dissipation (QCM-D) (Q-Sense AB, Sweden) monitoring on a Q-Sense E4 system to observe protein adsorption on a polymer brush. Each sample was analyzed in triplicate under a constant flow rate of 180 µl/min. The experimental procedure involved sequential steps: stabilizing the system with a 20 min injection of PBS, followed by the injection of Dulbecco's Modified Eagle's Medium (DMEM) (Thermo Fisher Scientific, USA) with GlutaMax, 10 % FBS, non-essential AA's, vitamins, and 25 mM HEPES for 20 min. Subsequently, the system was washed thoroughly with PBS for 20 min. Temperature was maintained at 25 °C throughout all measurements. Each sensor crystal was evaluated across various overtones (specifically the 3$^{rd}$, 5$^{th}$, 7$^{th}$, 9$^{th}$, and 11$^{th}$).

For rigid layers, the Sauerbrey equation was used to calculate the change in adsorbed mass (Δm) during film assembly, expressed as $\Delta m = -C \times \Delta f / n$, where C is a constant (17.7 $ng \cdot cm^{-2} \cdot Hz^{-1}$), Δf denotes the frequency change, and n represents the overtone used. The rigidity of these layers was confirmed by analyzing $\Delta f/n$ and $\Delta D$ responses, both of which indicated the presence of thin, rigid films. The determination relied on the ratio $\Delta D/(-\Delta f/n)$, where a value below $0.4 \times 10^{-6}\ Hz^{-1}$ suggests films are well-described by the Sauerbrey equation's model.[5]

## 8. *Bacterial cultivation*

The *Staphylococcus epidermidis* ATCC 35984 and *Pseudomonas aeruginosa* PAO1

ATCC 15692 used in this study were obtained from the DSMZ-German Collection of Microorganisms and Cell Culture GmbH, Germany. Overnight cultures were prepared in 50 mL Erlenmeyer flasks by transferring a single colony to 10 mL of tryptone soy broth (TSB) (Sigma-Aldrich, Germany) for *S. epidermidis* or to Lysogeny broth (LB) (Sigma-Aldrich, Germany) for *P. aeruginosa*. The cultures were incubated overnight at 37 °C with shaking at 100 RPM. Bacterial cells were harvested through centrifugation at 5000 RPM for 5 minutes and adjusted to a dilution of 0.43 OD at 600 nm in fresh PBS for flow experiments or fresh medium broth for biofilm growth experiments.

### *9. Bacterial adhesion and biofilm growth*

*S. epidermidis* or *P. aeruginosa* deposition on coated surfaces was observed using a Nikon Eclipse TE2000 microscope in bright-field transmission imaging mode with a 40x objective (Nikon Europe B.V, Austria). A sticky 0.2-mm bottomless microscope slide (Ibidi GmbH, Germany) equipped with Luer adaptors was used. All tubes and the flow chamber (height: 0.2 mm and length: 50 mm) were filled with sterile PBS, ensuring the elimination of air bubbles at a controlled shear rate of 0.5 ml/min. To study bacterial adhesion, a bacterial suspension in PBS was perfused through the chamber for 2 h, allowing real-time evaluation of adhering bacteria per unit area. Upon completion of the adhesion phase, the flow was switched to sterile PBS to rinse out the loosely bound bacteria from the surface and bacterial suspension from the chamber. Subsequently, a sterile culture medium (TSB or LB) was injected and allowed to flow for 20 h at a rate of 0.35 ml/min to study the biofilm formation.

During the bacterial adhesion study, images were captured every 5 min over 2 h, while for the overnight biofilm growth experiments, images were captured every 20 min. The number of adherent bacteria per unit area was quantified using ImageJ software (U.S. National Institutes of Health, USA).

For the biofilm growth under static conditions, surfaces were inoculated with *S. epidermidis* or *P. aeruginosa* in Petri dishes and then incubated at 37 °C for 24 h. Subsequently, surfaces were rinsed with PBS, and biofilms were imaged using a CCD camera attached to a phase-contrast microscope with a 40× objective. To assess bacterial viability, biofilms were stained with LIVE/DEAD (3.34 mM SYTO 9 and 2 mM propidium iodide, Invitrogen, Thermo Fischer Scientific, Austria) staining solution in PBS as previously outlined.[5]

## *10. Mouse monocytes J774A.1 and SaOS-2 osteosarcoma cell culturing and harvesting*

The SaOS-2 Osteosarcoma cells ACC 243 (DSMZ-German Collection of Microorganisms and Cell Culture GmbH, Germany) and Mouse macrophages (J774A.1 cell line) (DSMZ-German Collection of Microorganisms and Cell Culture GmbH, Germany) were cultured in DMEM supplemented with McCoy's 5A Medium with GlutaMAX (Thermo Fisher Scientific, Austria), 15% fetal bovine serum (FBS), and 1% Penicillin-Streptomycin (PenStrep) (Thermo Fisher Scientific, Austria). The cells were cultured in T75 cell culture flasks at 37 °C in a 5% $CO_2$ humidified environment until a confluency of 95% was reached. Cells were detached using TrypLE (Thermo Fischer Scientific, USA), and sedimented at 200 × g. Cells were harvested, stained with Trypan blue, counted using a Countess® automated cell counter (Invitrogen, Thermo Fisher Scientific, Austria), and diluted to the needed concentration in DMEM with GlutaMAX, 10% FBS, 25 mM HEPES (Thermo Fisher Scientific, Austria), and 1% Penicillin-Streptomycin.

## *11. Macrophage migration and phagocytic activity*

To study macrophage-bacterial interactions on surfaces, an IBIDI bottom coverslip with a flow chamber maintained at 37 °C was used. Prior to each experiment, all tubes and the flow chamber were filled with sterile PBS. The system was then primed with *S. epidermidis* in PBS, flowing at a shear rate of 0.5 ml/min. Subsequently, a suspension of J774A.1 ($2.5 \times 10^6$) DMEM with GlutaMAX, 10% FBS, and 25 mM HEPES without antibiotics was introduced. Interactions between bacteria and J774A.1 macrophages on the surfaces were observed using a phase-contrast microscope at 40× magnification. Images were collected throughout the 120 min assay at 1 min intervals.

As a control, substrates infected with *S. epidermidis* were injected with DMEM without antibiotics, allowing observation of bacterial growth in the absence of J774A.1 to assess how bacteria proliferate under the same conditions without the presence of immune cells. Additionally, separate experiments were conducted to observe the behavior of J774A.1 in sterile conditions without bacterial presence, providing a baseline for comparison.

## *12. SaOS-2 osteosarcoma cell attachment and spreading*

SaOS-2 cell suspension ($2.5 \times 10^6$ cells/mL) in DMEM- with GlutaMAX, 10% FBS, and 25 mM HEPES medium was injected into the microfluidic system and incubated at 37 °C for 90 min. Bright-field images were taken every 5 min using a 20× objective throughout the

90-min assay. Sterile DMEM medium was then injected and left for 22 h at a flow rate of 5 μl/min for the flow experiments.

Subsequently, the cells were fixed with Roti Histofix (ROTH, Germany) for 10 min, followed by a thorough rinse with PBS. Next, the cells were exposed to a 0.5% Triton X-100 solution (Sigma Aldrich, Germany) in PBS (1 mL per well) for 3 min and then rinsed with PBS three subsequent times and stained for 30 min with a solution consisting of 1% DAPI (Sigma Aldrich, Germany) and 2 μg/ml TRITC-phalloidin (Sigma Aldrich, Germany) in PBS. Following this staining process, the cells were rinsed with PBS and observed under a fluorescence microscope. The surface coverage by SaOS-2 cells was quantified using the ImageJ software.

**Table S1**. The average effective thickness and swelling ratio of co-polymer on gold-coated QCM crystals measured by spectroscopic ellipsometry. Each reported thickness represents the measurement of 3 coated substrates.

| **Sample** | **Silicon wafers** | **Gold-coated QCM crystals** | | |
|---|---|---|---|---|
| | Dry state (nm) | Dry state (nm) | Wet state (nm) | Swelling ratio |
| $pHP_{(97)}SB_{(3)}$ | 15.5 ± 2.4 | 26.6 ± 4.1 | 65.7 ± 2.9 | 2.5 ± 0.1 |
| $pCB_{(20)}HP_{(77)}SB_{(3)}$ | 22.2 ± 1.4 | 24.7 ± 3.0 | 54.3 ± 4.5 | 2.2 ± 0.0 |
| $pCB_{(30)}HP_{(67)}SB_{(3)}$ | 30.1 ± 3.4 | 29.8 ± 5.6 | 78.7 ± 7.8 | 2.6 ± 0.1 |

**Table S2.** The measured atomic concentration of homopolymer and copolymer brush samples.

| | **Atomic concentration, at.%** | | | | |
|---|---|---|---|---|---|
| **Sample** | C | O | N | S | Si |
| **Non-coated glass** | 13.8 | 50.9 | 0.9 | 0.0 | 26.0 |
| **pHPMAA** | 62.5 | 22.5 | 8.8 | 0.0 | 6.2 |
| **pCBMAA** | 71.0 | 16.8 | 11.2 | 0.0 | 1.0 |
| **pSBMAA** | 65.1 | 20.2 | 9.6 | 5.1 | 0.0 |
| $\mathbf{pHP_{(97)}SB_{(3)}}$ | 68.4 | 19.5 | 8.9 | 0.8 | 2.4 |
| $\mathbf{pCB_{(20)}HP_{(77)}SB_{(3)}}$ | 69.8 | 17.6 | 10.4 | 0.7 | 1.5 |
| $\mathbf{pCB_{(30)}HP_{(67)}SB_{(3)}}$ | 70.4 | 17.2 | 10.5 | 0.8 | 1.1 |

**Table S3.** Surface characterization using dynamic water contact angle.

| **Sample** | $\mathbf{\Theta_a}$ **[deg]** | $\mathbf{\Theta_r}$ **[deg]** |
|---|---|---|
| Non-coated glass | 81.6 ± 1.7 | 16.9 ± 0.6 |
| $pHP_{(97)}SB_{(3)}$ | 51.9 ± 1.6 | 19.0 ± 2.0 |
| $pCB_{(20)}HP_{(77)}SB_{(3)}$ | 49.8 ± 2.4 | 13.7 ± 0.1 |
| $pCB_{(30)}HP_{(67)}SB_{(3)}$ | 50.0 ± 2.6 | 10.7 ± 1.8 |

**Table S4.** ζ-potential of polymer brush-coated surfaces at pH 7.4.

| **Sample** | **ζ-potential before RGD binding [mV]** | **ζ-potential after RGD binding[mV]** | **ζ-potential blank [mV]** |
|---|---|---|---|
| $pHP_{(97)}SB_{(3)}$ | -9.19 ± 1.16 | -16.43 ± 1.15 | - |
| $pCB_{(20)}HP_{(77)}SB_{(3)}$ | -13.38 ± 1.77 | -2.68 ± 2.37 | -14.99 ± 1.24 |
| $pCB_{(30)}HP_{(67)}SB_{(3)}$ | -23.45 ± 4.90 | -7.05 ± 3.88 | - |

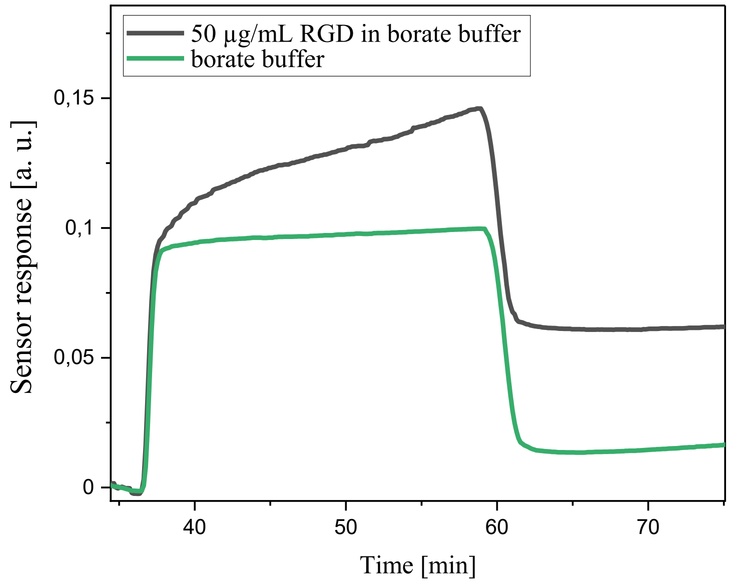


**Figure S2.** Representative SPR sensor response comparison of 50 µg/mL RGD in borate buffer vs. pure borate buffer on pCB$_{(20)}$HP$_{(77)}$SB$_{(3)}$.

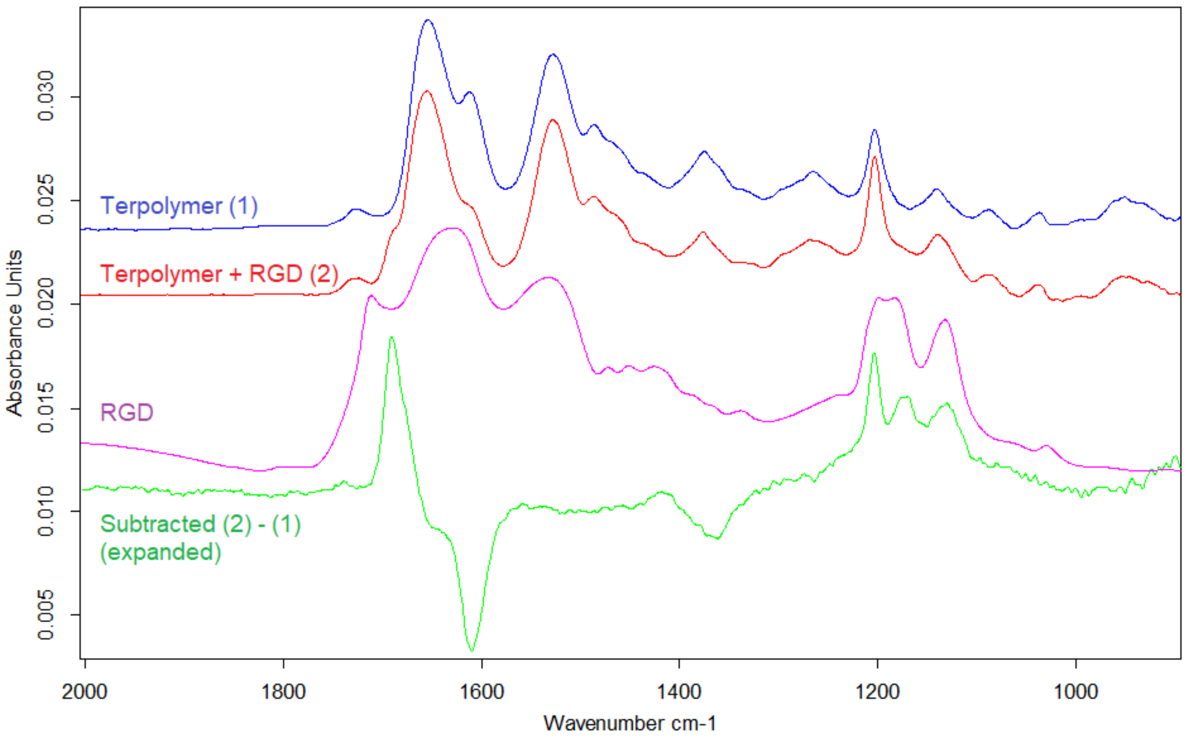


**Figure S3.** The IRRAS spectra of RDG binding to the terpolymer brush. The reference RGD spectrum was obtained by evaporating a drop of RGD solution on gold.

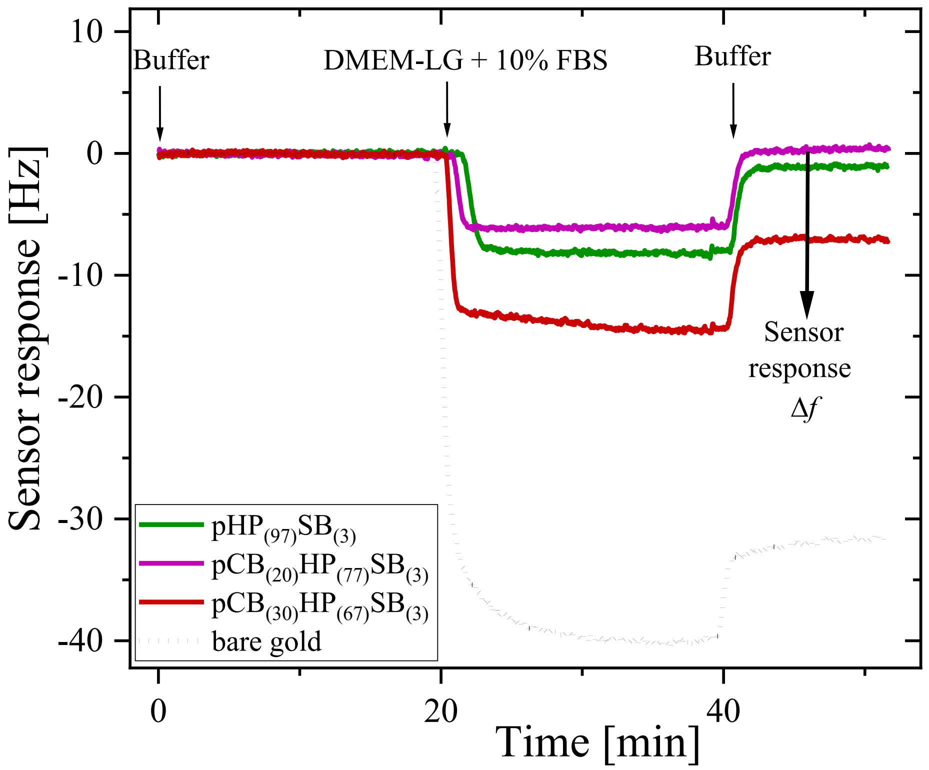


**Figure S4.** Representative QCM-D sensorgrams showing kinetics upon injection of cell culture medium (DMEM-LG + 10% FBS) onto surfaces with varying CBMAA content and bare gold QCM-D crystals.

**Table S5.** Comparative protein adsorption values on various coatings.

| **Coating** | **Protein Adsorption** | **Technique** | **Study** |
|---|---|---|---|
| p(CBMAA 20 mol%-*co*-HPMAA 77 mol%-*co*-SBMAA 3 mol%) | 12.6 ng/cm² for DMEM + 10% FBS | QCM-D | Our Study |
| p(CBMAA 15 mol%-*co*-HPMAA 82 mol%-*co*-SBMAA 3 mol%); pCBAA | 5.3 ng/cm²; 14.6 ng/cm² for undiluted human plasma | SPR | Forinova et al. (2021)[6] |
| pCBMA | 0.3 ng/cm² for 1.0 mg/ml fibrinogen | SPR | Cheng et al. (2009)[7] |
| DOPA₂–pCBMA₂ | 6.3 ng/cm² for undiluted human plasma; 11.7 ng/cm² from serum | SPR | Brault et al. (2010)[8] |
| pSBMAA300-catechol | 10.5 ng/cm² for 10% human serum | SPR | Li et al. (2008)[9] |
| pSBMA | Low (specific values not provided) for 1.0 mg/ml fibrinogen | SPR | Cheng et al. (2007)[10] |

| | | | |
|---|---|---|---|
| pCBAA; pCBMAA; p(CBMAA 65%-*co*-HPMAA 35%); p(CBMAA 35 mol%-*co*-HPMAA 65 mol%); pHPMAA | 0 ng/cm² for 100% human blood plasma/cell-free culture medium | SPR | Visova et al, (2020)[3] |
| Poly *N*,*N*-dimethyl lactamide acrylate; pHPMAA | Low (specific values not provided) for undiluted human plasma | SPR | Englert et al, (2023)[11] |
| Hierarchical zwitterionic polymer brushes | 12 ng/cm² to BSA, aprox 150 ng/cm² to 100% human blood plasma | QCM-D | Teunissen et al, (2022)[12] |
| PSEMA–PSBMA–PSEMA triblock copolymer; PSBMA–PSEMA block copolymer | Excellent resistance (specific values not provided) to BSA adsorption | QCM-D | Xia et al, (2023)[13] |
| poly(2-ethyl-2-oxo-1,3,2-dioxaphospholane) | 17 ng/cm² for diluted human serum | QCM-D | Perez et al, (2024)[14] |

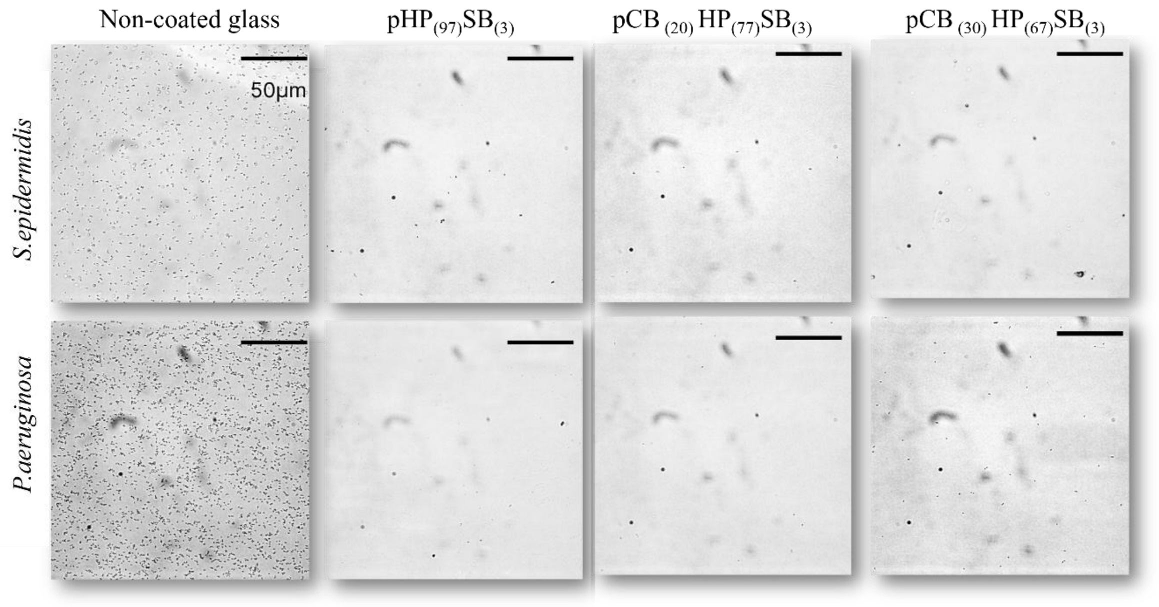


**Figure S5.** Representative microscope images of adherent bacteria and biofilm formation after 2 h.

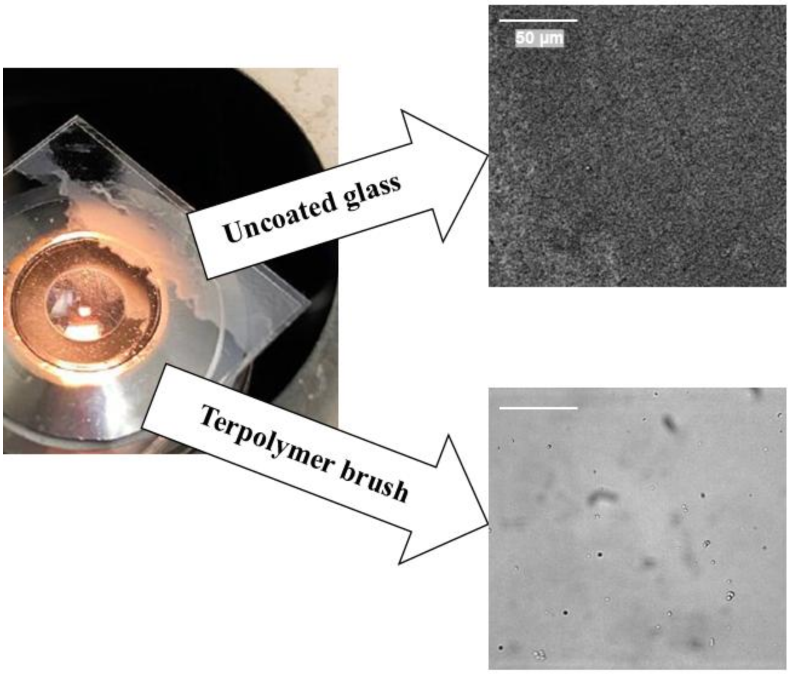


**Figure S6.** Representative phase-contrast microscope images illustrating *S. epidermidis* biofilm formation under static conditions on glass coverslips after 24 hours of incubation at 37 °C in TSB. The images compare biofilm development on uncoated glass versus surfaces coated with the terpolymer brush $pCB_{(20)}HP_{(77)}SB_{(3)}$. The long-term antifouling efficacy of $pCB_{(20)}HP_{(77)}SB_{(3)}$ was further validated under static conditions by incubating coated and uncoated surfaces in TSB at 37 °C for 24 h within the same Petri dish. A striking contrast was observed: while uncoated glass exhibited extensive biofilm formation, the terpolymer-coated areas remained free of bacterial colonization, demonstrating the material's strong antifouling properties in a stationary environment, independent of flow dynamics.

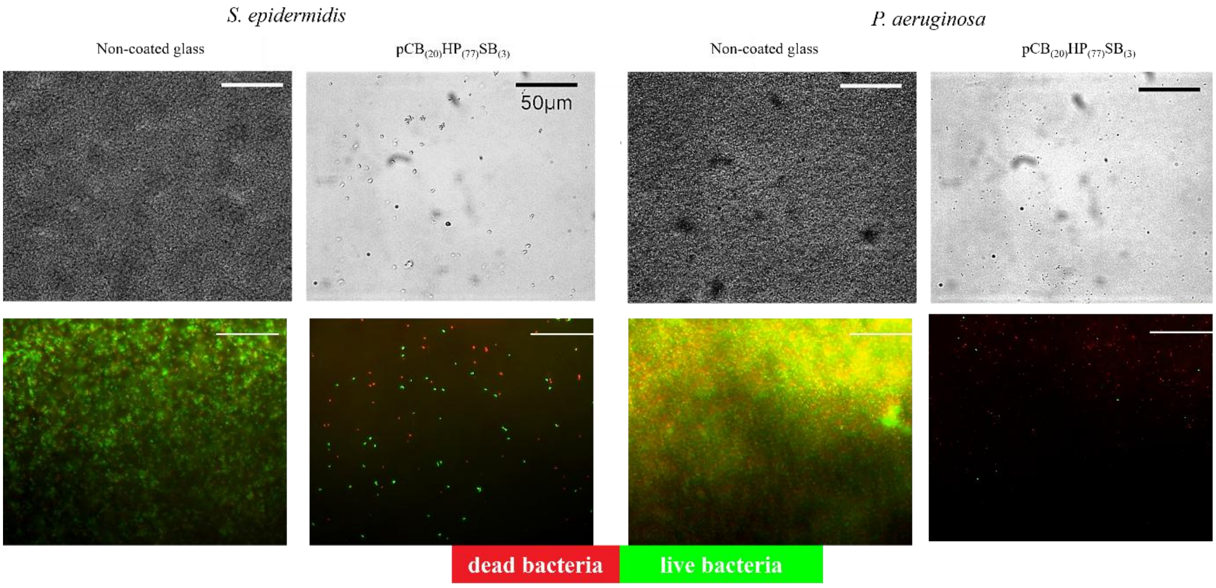


**Figure S7.** Representative phase-contrast and fluorescence microscope images of static biofilm growth of *S. epidermidis* and *P. aeruginosa* after incubation at 37 °C for 24 h on the uncoated glass coverslip as control and terpolymer-grafted glass coverslip surfaces pCB$_{(20)}$HP$_{(77)}$SB$_{(3)}$. Bacteria were stained using vitality staining solution (3.34 mM SYTO 9 and 20 mM propidium iodide in PBS) and were incubated for 15 min in the dark at room temperature.

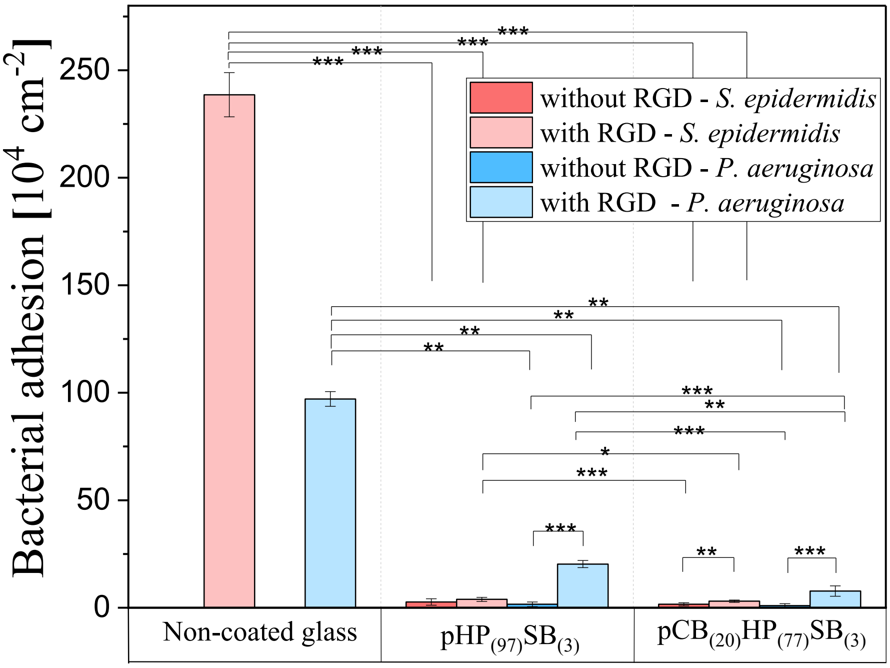


**Figure S8.** Bacterial adhesion on uncoated and polymer brush-grafted glass coverslips with and without immobilized RGD-peptide over 2 h.

**Table S6.** Comparative bacterial adhesion on various coatings.

| Coating | Result | Study |
|---|---|---|
| p(CBMAA 20 mol%-*co*-HPMAA 77 mol%-*co*-SBMAA 3 mol%) | 2.7 x $10^4$ bacteria/cm² for *S. epidermidis;* 2.3 x $10^4$ bacteria/cm² for *P. aeruginosa* after 20 h | Our Study |
| p(2-ethyl-2-oxo-1,3,2-dioxaphospholane) | 97.5% reduction for *S. aureus*; 98.8% for *E. coli;* no biofilm formation at 22 h | Perez et al, (2024)[14] |
| pSBMA300-catechol | bacterial counts being very low (~$10^6$ cells/mm²) after 24 hours | Li et al. (2008)[9] |
| pSBMA | no biofilm formation for *S. epidermidis* and *P. aeruginosa* at 24 h | Cheng et al. (2007)[10] |
| photo-cross-linked zwitterionic polymer brushes supported by poly(ether ether ketone) substrate | Low (specific values not provided) *E. coli* adhesion after 6 h | Nakano et al, (2020)[15] |
| Poly *N*,*N*-dimethyl lactamide acrylate | Low (specific values not provided) *E. coli* adhesion after 24 h | Englert et al, (2023)[11] |
| pSEMA–pSBMA–pSEMA triblock copolymer; pSBMA–pSEMA block copolymer | 99.6% reduction for *S. aureus*; 98.9% for *E. coli* after 3 h | Xia et al, (2023)[13] |
| amino acid-based zwitterionic polymers | Low (specific values not provided) *P. aeruginosa* adhesion after 24 h | Liu et al, (2016)[16] |
| p(CBMAA-*co*-acrylic acid) | Low (specific values not provided) for *S. aureus* and *E. coli* after 24 hours | Hassani et al, (2024)[17] |
| carboxybetaine functionalized polysiloxanesstarts | started from $10^5$ bacteria/cm$^2$ after 24 hours for *E. coli* | Cheng et al, (2014)[18] |

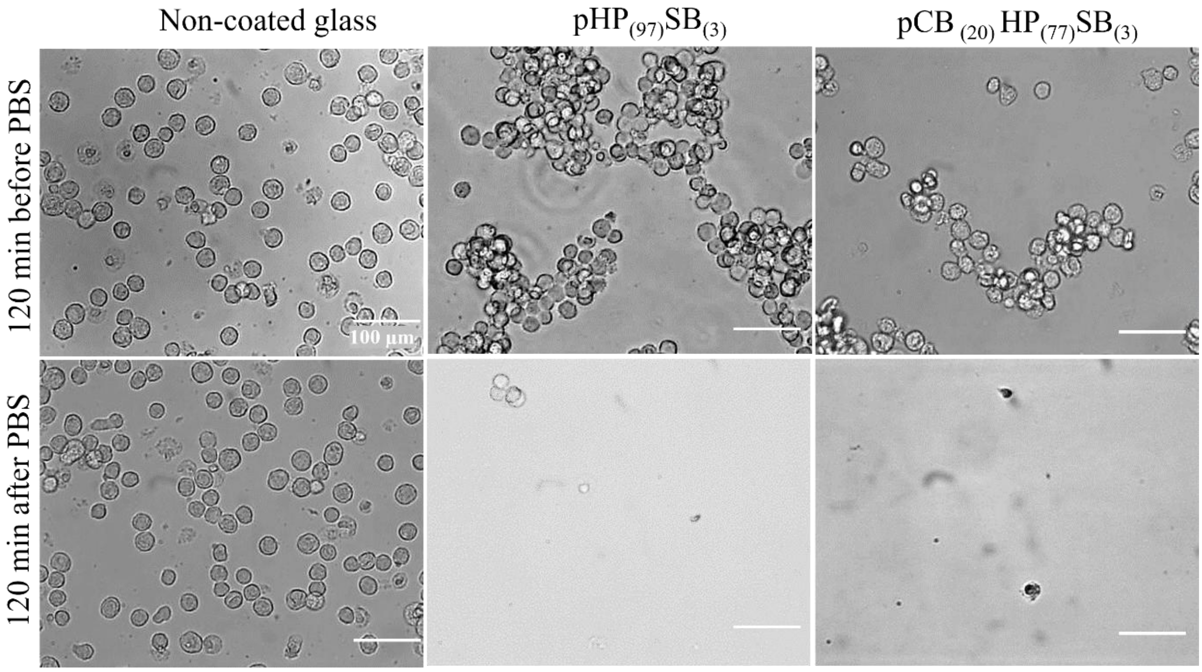


**Figure S9.** Representative phase-contrast images illustrating the morphologies of macrophages adhering to different coatings after 120 min incubation of SaOS-2 monoculture without bacteria, following PBS washing for control measurements. The images compare variations in macrophage adhesion and distribution across coated and uncoated surfaces, highlighting differences in cell clustering and surface coverage. The scale bar represents 100 µm.

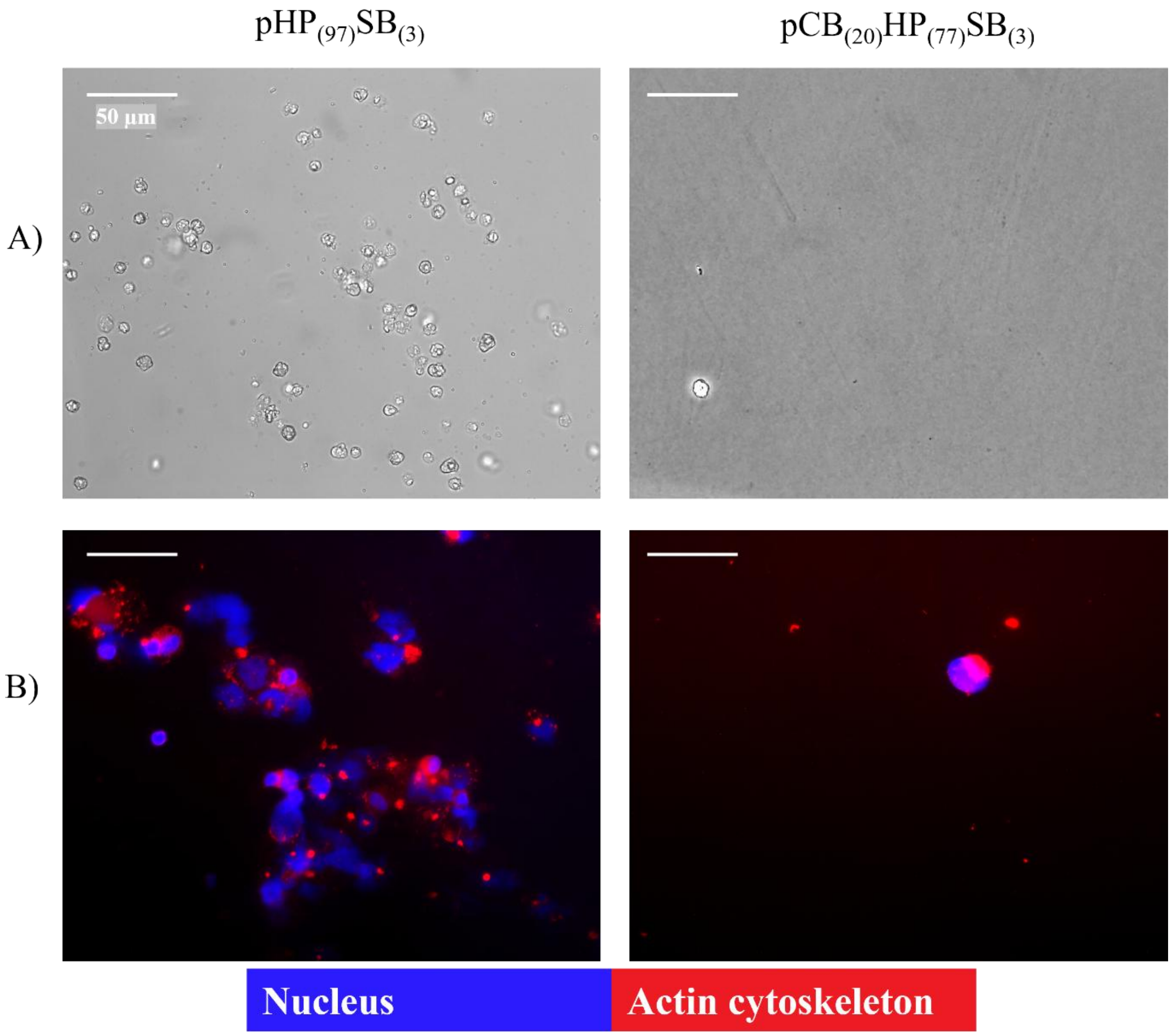


**Figure S10.** A) Bright-field microscopy and B) merged fluorescence images of SaOS-2 cell attachment and spreading after 24 h on polymer brush-coated glass. The scale bar is 50 µm.

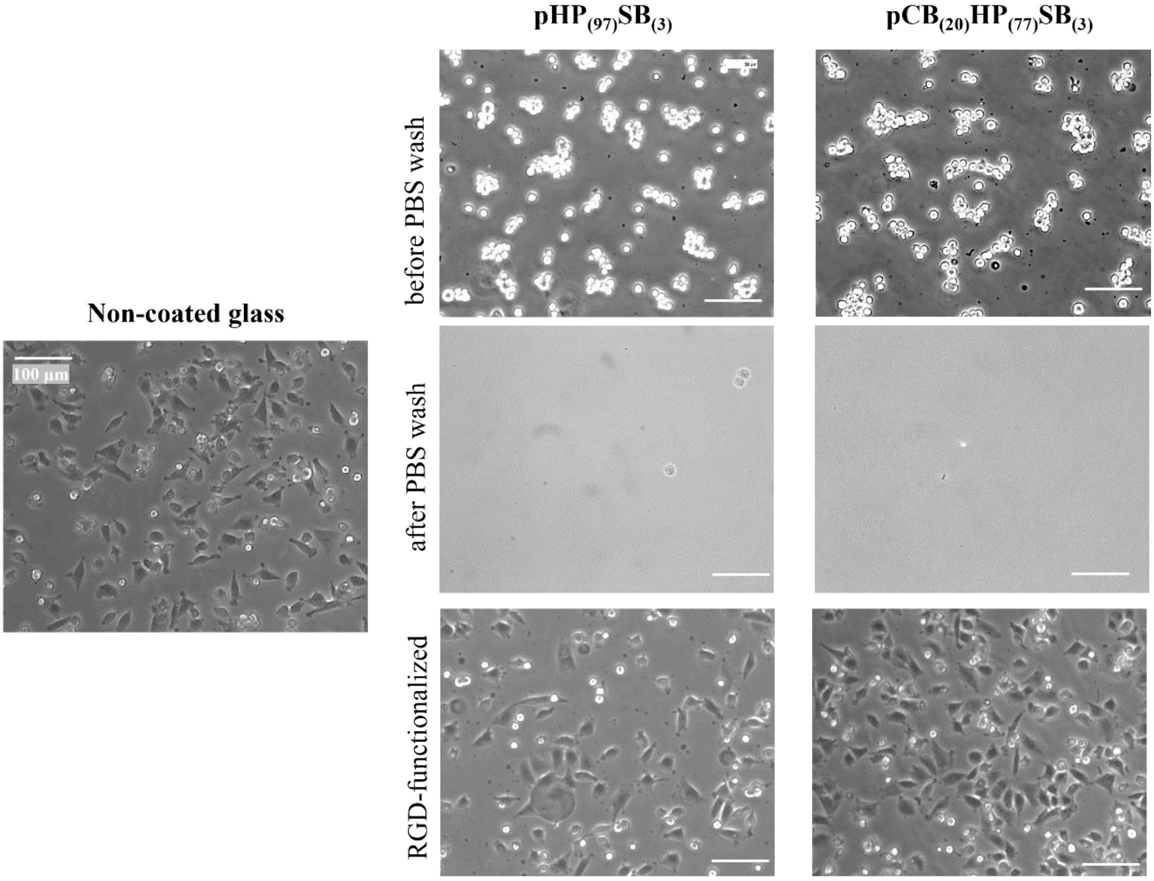


**Figure S11.** Representative phase-contrast microscopic images illustrating the morphologies of SaOS-2 adhering to different coatings after 120 min, with and without RGD peptide immobilization. The scale bar is 100 μm.

## References


(1) Forinová, M.; Pilipenco, A.; Lynn, N. S.; Obořilová, R.; Šimečková, H.; Vrabcová, M.; Spasovová, M.; Jack, R.; Horák, P.; Houska, M.; et al. A reusable QCM biosensor with stable antifouling nano-coating for on-site reagent-free rapid detection of *E. coli* O157:H7 in food products. *Food Control* **2024**, *165*, 110695, DOI: 10.1016/j.foodcont.2024.110695.
(2) Covato, C.; Pilipenco, A.; Scheberl, A.; Reimhult, E.; Subbiahdoss, G. Osteoblasts win the race for the surface on DNA polyelectrolyte multilayer coatings against S. epidermidis but not against *S. aureus*. *J. Polym. Sci. B Polym. Phys.* **2025**, *245*, 114336, DOI: 10.1016/j.colsurfb.2024.114336
(3) Víšová, I.; Smolková, B.; Uzhytchak, M.; Vrabcová, M.; Zhigunova, Y.; Houska, M.; Surman, F.; de los Santos Pereira, A.; Lunov, O.; Dejneka, A.; et al. Modulation of Living Cell Behavior with Ultra-Low Fouling Polymer Brush Interfaces. *Macromol. Biosci.* **2020**, *20* (3), 1900351, DOI: 10.1002/mabi.201900351.
(4) Víšová, I.; Houska, M.; Spasovová, M.; Forinová, M.; Pilipenco, A.; Mezuláníková, K.; Tomandlová, M.; Mrkvová, K.; Vrabcová, M.; Dejneka, A.; et al. Tuning of Surface Charge of Functionalized Poly(Carboxybetaine) Brushes Can Significantly Improve Label-Free Biosensing in Complex Media. *Adv. Mater. Interfaces* **2022**, *9* (33), 2270182, DOI: 10.1002/admi.202270182.
(5) Ouni, O. A.; Subbiahdoss, G.; Scheberl, A.; Reimhult, E. DNA polyelectrolyte multilayer coatings are antifouling and promote mammalian cell adhesion. *Materials* **2021**, *14* (16), 4596, DOI: 10.3390/ma14164596.
(6) Forinová, M.; Pilipenco, A.; Víšová, I.; Lynn, N. S.; Dostálek, J.; Mašková, H.; Hönig, V.; Palus, M.; Selinger, M.; Kočová, P.; et al. Functionalized terpolymer-brush-based biointerface with improved antifouling properties for ultra-sensitive direct detection of virus in crude clinical samples. *ACS Appl. Mater. Interfaces* **2021**, DOI: 10.1021/acsami.1c16930.
(7) Cheng, G.; Li, G.; Xue, H.; Chen, S.; Bryers, J. D.; Jiang, S. Zwitterionic carboxybetaine polymer surfaces and their resistance to long-term biofilm formation. *Biomaterials* **2009**, *30* (28), 5234-5240, DOI: 10.1016/j.biomaterials.2009.05.058.
(8) Brault, N. D.; Gao, C.; Xue, H.; Piliarik, M.; Homola, J.; Jiang, S.; Yu, Q. Ultra-low fouling and functionalizable zwitterionic coatings grafted onto SiO2 via a biomimetic adhesive group for sensing and detection in complex media. *Biosens. Bioelectron.* **2010**, *25* (10), 2276-2282, DOI: 10.1016/j.bios.2010.03.012.
(9) Li, G.; Cheng, G.; Xue, H.; Chen, S.; Zhang, F.; Jiang, S. Ultra low fouling zwitterionic polymers with a biomimetic adhesive group. *Biomaterials* **2008**, *29* (35), 4592-4597, DOI: 10.1016/j.biomaterials.2008.08.021.
(10) Cheng, G.; Zhang, Z.; Chen, S.; Bryers, J. D.; Jiang, S. Inhibition of bacterial adhesion and biofilm formation on zwitterionic surfaces. *Biomaterials* **2007**, *28* (29), 4192-4199, DOI: 10.1016/j.biomaterials.2007.05.041.
(11) Englert, J.; Palà, M.; Witzdam, L.; Rayatdoost, F.; Grottke, O.; Lligadas, G.; Rodriguez-Emmenegger, C. Green Solvent-Based Antifouling Polymer Brushes Demonstrate Excellent Hemocompatibility. *Langmuir* **2023**, *39* (50), 18476-18485, DOI: 10.1021/acs.langmuir.3c02765.
(12) Teunissen, L. W.; Kuzmyn, A. R.; Ruggeri, F. S.; Smulders, M. M. J.; Zuilhof, H. Thermoresponsive, Pyrrolidone-Based Antifouling Polymer Brushes. *Adv. Mater. Interfaces* **2022**, *9* (6), 2101717, DOI: 10.1002/admi.202101717.
(13) Xia, X.; Yuan, X.; Zhang, G.; Su, Z. Antifouling Surfaces Based on Polyzwitterion Loop Brushes. *Acs Appl. Mater. Inter.* **2023**, *15* (40), 47520-47530, DOI: 10.1021/acsami.3c10267.
(14) Pérez, M. B.; Resendiz-Lara, D. A.; Matsushita, Y.; Kakinoki, S.; Iwasaki, Y.; Hempenius, M. A.; de Beer, S.; Wurm, F. R. Creating Anti-Biofouling Surfaces by

Degradable Main-chain Polyphosphoester Polymer Brushes. *Adv. Funct. Mater.* **2024**, *34* (32), 2316201, DOI: 10.1002/adfm.202316201.
(15) Nakano, H.; Noguchi, Y.; Kakinoki, S.; Yamakawa, M.; Osaka, I.; Iwasaki, Y. Highly Durable Lubricity of Photo-Cross-Linked Zwitterionic Polymer Brushes Supported by Poly(ether ether ketone) Substrate. *ACS Appl. Bio Mater.***2020**, *3* (2), 1071-1078, DOI: 10.1021/acsabm.9b01040.
(16) Liu, Q.; Li, W.; Wang, H.; Newby, B.-m. Z.; Cheng, F.; Liu, L. Amino Acid-Based Zwitterionic Polymer Surfaces Highly Resist Long-Term Bacterial Adhesion. *Langmuir* **2016**, *32* (31), 7866-7874, DOI: 10.1021/acs.langmuir.6b01329.
(17) Hassani, M.; Kamankesh, M.; Rad-Malekshahi, M.; Rostamizadeh, K.; Rezaee, F.; Haririan, I.; Daghighi, S. M. Biomaterials coated with zwitterionic polymer brush demonstrated significant resistance to bacterial adhesion and biofilm formation in comparison to brush coatings incorporated with antibiotics. *Colloids Surf., B* **2024**, *234*, 113671, DOI: 10.1016/j.colsurfb.2023.113671.
(18) Cheng, L.; Liu, Q.; Lei, Y.; Lin, Y.; Zhang, A. The synthesis and characterization of carboxybetaine functionalized polysiloxanes for the preparation of anti-fouling surfaces. *RSC Adv.* **2014**, *4* (97), 54372-54381, DOI: 10.1039/C4RA09171J.
